# Polyphosphate and tyrosine phosphorylation in the N-terminal domain of the human mitochondrial Lon protease disrupts its functions

**DOI:** 10.1038/s41598-024-60030-9

**Published:** 2024-04-30

**Authors:** Nina Kunová, Gabriela Ondrovičová, Jacob A. Bauer, Veronika Krajčovičová, Matyáš Pinkas, Barbora Stojkovičová, Henrieta Havalová, Veronika Lukáčová, Lenka Kohútová, Július Košťan, Lucia Martináková, Peter Baráth, Jiří Nováček, Sebastian Zoll, Sami Kereïche, Eva Kutejová, Vladimír Pevala

**Affiliations:** 1 Department of Biochemistry and Protein Structure, Institute of Molecular Biology, Slovak Academy of Sciences, Dúbravská Cesta 21, 845 51 Bratislava, Slovakia; 2 Institute of Biology and Medical Genetics, First Faculty of Medicine, Charles University in Prague, Prague, Czech Republic; 3 CEITEC, Masaryk University in Brno, Brno, Czech Republic; 4 Medirex Group Academy, Nitra, Slovakia; 5 Institute of Chemistry, Slovak Academy of Sciences, Bratislava, Slovakia; 6 Department of Structural and Computational Biology, Max Perutz Labs, University of Vienna, Campus Vienna, Biocenter 5, 1030 Vienna, Austria; 7 Institute of Organic Chemistry and Biochemistry of the Czech Academy of Sciences, Flemingovo Namesti 542/2, 16000 Prague, Czech Republic; 8 Present Address: Laboratory of Clinical and Molecular Genetics, National Institute of Children’s Diseases, Limbová 1, 833 40 Bratislava, Slovakia

**Keywords:** Electron microscopy, Mitochondrial proteins, Phosphoproteins, Proteases, Proteomic analysis, Chromatography, Electrophoresis, Protein purification, Western blot, Mass spectrometry, Phosphorylation, Mitochondria

## Abstract

Phosphorylation plays a crucial role in the regulation of many fundamental cellular processes. Phosphorylation levels are increased in many cancer cells where they may promote changes in mitochondrial homeostasis. Proteomic studies on various types of cancer identified 17 phosphorylation sites within the human ATP-dependent protease Lon, which degrades misfolded, unassembled and oxidatively damaged proteins in mitochondria. Most of these sites were found in Lon’s N-terminal (NTD) and ATPase domains, though little is known about the effects on their function. By combining the biochemical and cryo-electron microscopy studies, we show the effect of Tyr186 and Tyr394 phosphorylations in Lon’s NTD, which greatly reduce all Lon activities without affecting its ability to bind substrates or perturbing its tertiary structure. A substantial reduction in Lon’s activities is also observed in the presence of polyphosphate, whose amount significantly increases in cancer cells. Our study thus provides an insight into the possible fine-tuning of Lon activities in human diseases, which highlights Lon’s importance in maintaining proteostasis in mitochondria.

## Introduction

Mitochondria are essential components of eukaryotic cells, which supply them with energy and contribute to many essential metabolic pathways. Here, like in any other cellular compartment, phosphorylation as one of the most extensively studied post-translational modifications (PTM) plays an important role in regulating any number of proteins by promoting their conformational changes, activating/deactivating enzymes, facilitating protein degradation, and mediating protein–protein interactions. In fact, it was found that ~ 40% of the mitochondrial proteome could be phosphorylated^1,2^, which in humans could lead to development of severe pathologies. Particularly in cancer, the level of protein phosphorylation often increases.

Mitochondria possess several protein quality control mechanisms that allow their quick adaptation to environmental changes. One of them, ATP-dependent protease Lon, is a member of the AAA^+^ family (ATPase associated with various cellular activities) and is responsible for selective degradation of unfolded, oxidatively damaged, and short-lived regulatory proteins, which prevents their toxic aggregation. This activity gives Lon a key role in the fine tuning of several cellular processes, which affect mitochondrial protein turnover, the regulation of mtDNA replication, cellular respiration, oxidative phosphorylation, and mitochondrial morphology and dynamics^3^.

Human Lon is ubiquitously expressed in various tissues and cell types with the highest levels observed in the adrenal glands, liver, brain, heart, skeletal muscle, and placenta^4^. In *Drosophila*, the overexpression of Lon destabilizes mtDNA, while its lower levels influence mtDNA transcription^5^. In several malignant cell lines, Lon levels were found to be increased^6^ and an increase in Lon was also observed when prostate adenocarcinoma PC3 and glioblastoma LN229 cells were exposed to hypoxia. In rhabdomyosarcoma cells, hypoxic conditions doubled the levels of Lon mRNA^7^, whereas in hepatocarcinoma cells, embryonic fibroblasts, and the liposarcoma SW872 cell line^8^, H_2_O_2_ exposure increased its levels up to fivefold. The higher levels of Lon were found to correlate with reduced overall survival in patients with neuroblastoma, breast and colon adenocarcinomas and renal cell carcinoma^9^.

Functional *h*Lon is structurally organized into homo-oligomers consisting of six^10^ identical subunits. Each subunit contains a cleavable mitochondrial targeting sequence (MTS), a substrate-binding N-terminal domain (NTD), a central ATPase domain (AAA^+^ module) with conserved Walker A and Walker B motifs, and a C-terminal proteolytic domain (CTD) with a serine-lysine (Ser855-Lys898) catalytic dyad in the active site^10,11^, all encoded in one polypeptide chain. Following its mitochondrial localization, Lon’s NTD specifically recognizes and binds protein substrates, which are then unfolded and translocated into the enzyme’s proteolytic chamber formed of the ATPase and protease domains, where they are degraded into small peptides^10,12,13^.

The cryo-electron microscopy (cryo-EM) structures reported recently^14–17^ show that the protease and ATPase domains of human Lon (*h*Lon) form a single functional unit (called the “head”^15^), while the NTD forms a separate and substantially more flexible domain (the “legs”). The junction between these two domains (the “neck”) was shown to be important for several of *h*Lon’s functions including its proper oligomerization^15,18^. Structurally, the neck is comprised of a triangular gate from the NTD side composed of three long α-helices and an entrance pore on the ATPase side, which the substrate passes through as it enters the ATPase-protease “head” domain.

**Figure 1:**
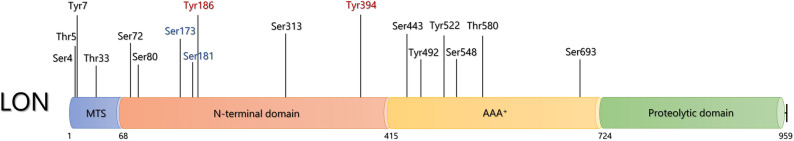
The currently known phosphorylation sites in the *h*Lon amino-acid sequence. Presently, 17 phosphorylation sites have been identified, including nine phosphorylated serines (Ser), five phosphorylated tyrosines (Tyr), and three phosphorylated threonines (Thr)^19^. Ser173 and Ser181 (colored blue) are phosphorylated by Ser/Thr kinase Akt1 (Protein kinase B, PKB), which accumulates during the hypoxia associated with cancer^9^. Tyr186 and Tyr394 (colored red) are studied in this paper.

According to the PhosphoSitePlus database^19^, *h*Lon can be phosphorylated on 17 amino-acid residues (Ser4, Thr5, Tyr7, Thr33, Ser72, Ser80, Ser173, Ser181, Tyr186, Ser313, Tyr394, Ser443, Tyr492, Ser548, Tyr552, Thr580, and Ser693). Most of these are located either in the N-terminal domain or in the ATPase module (AAA^+^), though four occur in Lon’s MTS (Fig. 1). Mass spectrometry (MS) analyses of various, mostly cancerous tissues found that Tyr394 residing in the “neck” region of the NTD is the most frequently phosphorylated residue in *h*Lon. This modification has been found in lung tumors^20^, sarcomas^21^, and gastrointestinal tumors, and also in cardiac diseases such as ventricular tachycardia and atrial fibrillation^19^, but there is presently no information on the influence of Tyr394 phosphorylation on the *h*Lon itself. Moreover, *h*Lon’s proteolytic activity can be enhanced by the phosphorylation at Ser173 and Ser181 mediated by the serine/threonine protein kinase Akt1^9^, which accumulates upon hypoxia in tumor-cell mitochondria^22^. Hypoxic conditions also lead to increased levels of Lon in human prostate adenocarcinoma and glioblastoma, but not in normal fibroblasts^9^. Phosphorylated *h*Lon could thus serve as an effector of mitochondrial reprogramming in cancer, which is known to exploit the mitochondrial protein quality control network to promote the malignant growth.

Interestingly, inorganic polyphosphate (polyP), a compound consisting of tens to hundreds of phosphates linked by high-energy phosphoanhydride bonds, like those found in ATP, is also upregulated in some cancer cells, including brain tumor initiating cells (BTICs), human bronchioloalveolar adenocarcinoma, invasive ductal adenocarcinoma, small intestine adenocarcinoma, prostate adenocarcinoma and medulloblastoma^23^. In these cells, it was found to be preferentially localized inside the mitochondria. Boyineni et al.^23^ hypothesized that polyP may play a role in cancer cells as an energy source similar to its role in bacteria during starvation or hypoxia. In *E. coli*, Lon protease was found to bind polyP and degrade ribosomal proteins S2, L9, and L13^24^. PolyP also stimulates the degradation of purified ribosomal proteins L9, L13, and L17, but not L15 or L18. In contrast, the degradation of the *E. coli* cell division inhibitor SulA fused to a maltose-binding protein was slightly inhibited when polyP was present. Therefore, polyP does not always enhance Lon-mediated protein degradation.

In this study, we investigated the effects that phosphorylating two Lon tyrosine residues, Tyr186 (found in leukemia cells^19^) and Tyr394 - the only two found in its NTD - might have on the structure and function of *h*Lon and the effect of polyphosphate on *h*Lon’s stability and activities. The N-terminal domain serves as the first contact point where the substrate initially binds to the Lon protease, leading to ATP hydrolysis and substrate translocation into the proteolytic cavity. Modification of this domain might result in significant changes to Lon’s activities. Tyr394 might especially be expected to have an effect since it is located at the gate through which the substrate must pass to travel between the NTD and the ATPase-Protease domain. Since *h*Lon represents a key component controlling mitochondrial homeostasis and it is exploited in many tumor cells^3,25^, our results could substantially contribute to better understanding the rearrangements of mitochondrial functions in cancer.

## Results

### Characterization of *h*Lon phosphorylation variants

To assess the effects of phosphorylations observed in cancer cells on *h*Lon functions, we mutated the human *LONP1* gene to encode phosphorylated or phosphorylation-mimicking variants of human mitochondrial Lon protease lacking the N-terminal MTS and fused to a hexahistidine tag (6 × His) using *E. coli* expression systems (Supplementary Table S2). Two *h*Lon mutants with the TAG *amber* stop codon were prepared for an orthogonal translation system in *E. coli* C321 (DE3) cells^26^, which were designed for the introduction of *para*-carboxymethyl-l-phenylalanine (pCMF), a non-hydrolysable analogue of phosphotyrosine^27^, to the protein of interest. In our case, pCMF was incorporated at positions 186 (pCMF186) and 394 (pCMF394) in the amino-acid sequence of *h*Lon (referred to hereafter as “phosphorylated” *h*Lon variants). Other *h*Lon mutants were produced by exchanging Tyr186 and Tyr394 for glutamates (Y186E; Y394E), thereby mimicking the negative charge introduced by phosphorylation (hereafter “phosphorylation-mimicking” *h*Lon variants), or for phenylalanines (Y186F; Y394F), which acted as non-phosphorylatable controls (these mutations were introduced to verify that the effects of phosphorylation were not due to the loss of functionality occasioned by the removal of the Tyr hydroxyl group). The His-tagged *h*Lon proteins were isolated using nickel affinity chromatography and further purified by size-exclusion chromatography (SEC) (Supplementary Figs. S1–S3). The proper introduction of pCMF at the sites of Tyr186 and Tyr394 in *h*Lon was confirmed by MS–MS analysis (Supplementary Table S3). Both SEC and glutaraldehyde crosslinking (Supplementary Fig. S4) confirmed that all *h*Lon versions formed oligomers in solution, which is an important prerequisite for proper *h*Lon functioning.

The thermal stability of the enzymes was checked using nanoDSF. For each *h*Lon variant, the denaturation temperature (T_m_), at which 50% of the protein unfolded, and the temperature at which aggregation began (T_onset_), were determined (Supplementary Figs. S5, S6 and Table S4). The denaturation temperature of wild-type *h*Lon (WT) was higher (50.5 °C) than that of all other variants, which ranged from 45.2 to 50.3 °C (Supplementary Table S4). Lower T_m_ normally implies lower protein stability, however the T_m_ differences of about 5 °C for the mutant *h*Lon variants were not great enough to cause substantial changes in the enzymes’ tendencies to unfold at the temperature *h*Lon normally acts in (37 °C). The onset of aggregation (T_onset_) for each *h*Lon variant ranged from about 48.6 to 59 °C (Supplementary Table S4) with peaks shifted to temperatures of at least 48 °C and more (Supplementary Figs. S5B, S6B) meaning that the enzymes should be fully functional for assays determined under more physiological conditions.

### The influence of phosphorylation on *h*Lon activities

**Figure 2:**
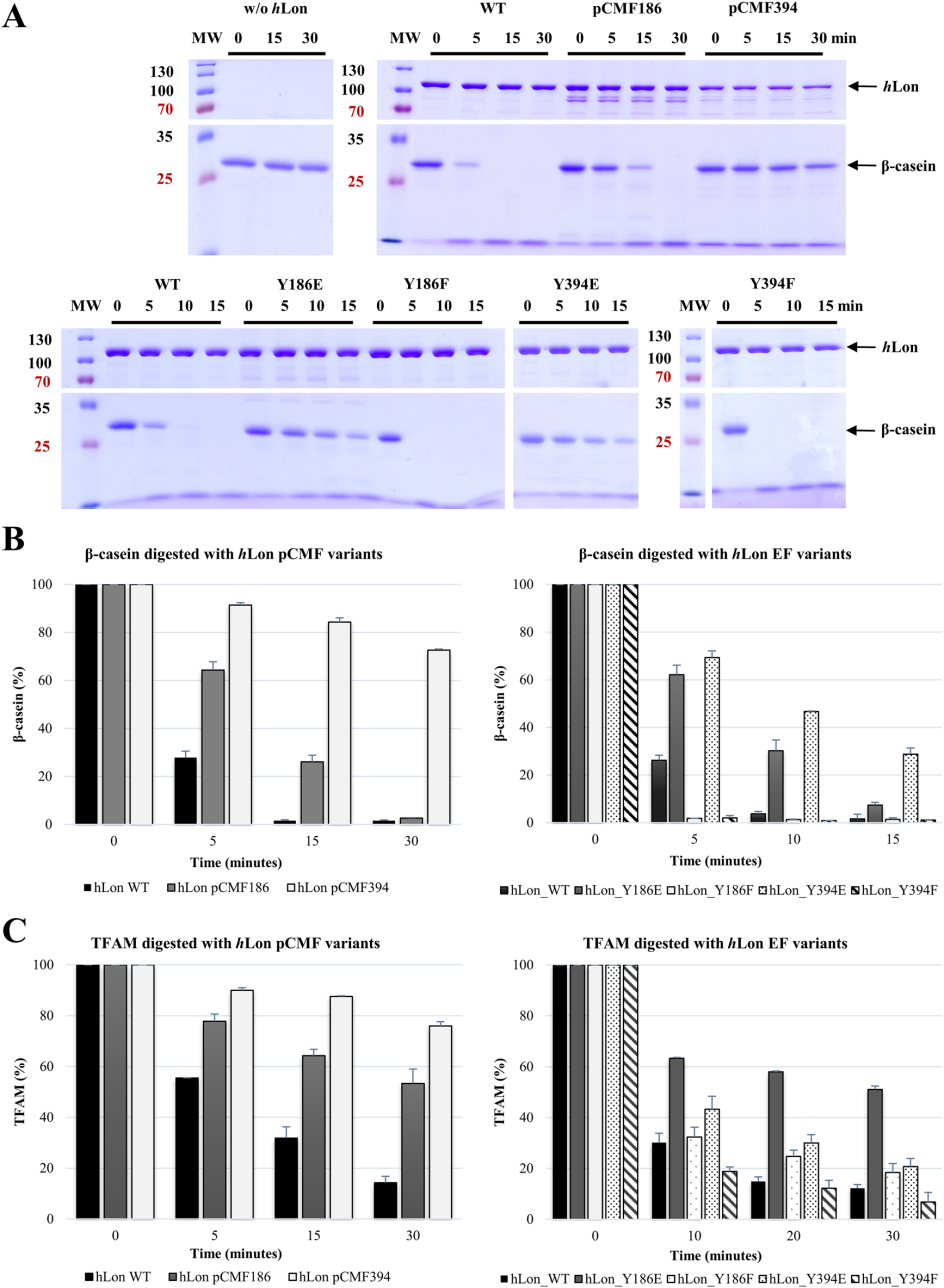
Protein substrates digested by the phosphorylated and phosphorylation-mimicking *h*Lon variants. (**A**) 1 μg β-casein or TFAM was incubated with 1 μg of the given *h*Lon variant in reactions containing 2 mM ATP and 10 mM MgCl_2_. Samples were withdrawn at the indicated times and loaded on a 12% SDS–polyacrylamide gels (only cropped images are shown here). (**B**) and (**C**) Quantification analyses of *h*Lon-digestion experiments. Band intensities were evaluated with ImageJ (v.1.53)^29^ separately for each studied protein. Each panel arises from the gels shown in (**A**) and Supplementary Fig. S7. The height of a given bar represents the mean value, and the error bars show ± one standard deviation from at least three separate measurements. *MW* - molecular weight marker; *WT -* wild-type *h*Lon; *pCMF186,* pCMF394 - phosphorylated *h*Lon variants; *Y186E,* Y394E - phosphorylation-mimicking *h*Lon mutants; *Y186F,* Y394F - control *h*Lon mutants.

The ability of *h*Lon to degrade protein substrates was tested by characterizing its proteolytic digestion of β-casein, as a universal protease substrate, and TFAM, as a physiological *h*Lon substrate^5,28^, in the presence of ATP and Mg^2+^ (Fig. 2 and Supplementary Fig. S7).

As can be seen, replacing either Tyr186 or Tyr394 with pCMF greatly reduces *h*Lon’s overall activity. Substrate degradation by *h*Lon_pCMF394 was particularly affected, with most of the protein substrate remaining undigested after 30 min. Replacing Tyr186 with pCMF also reduced substrate digestion, though not as dramatically as the pCMF394 substitution (Fig. 2 and Supplementary Fig. S7). Here, we also examined the effect of the bacterial expression system used for synthesizing the pCMF-modified *h*Lons by comparing the caseinolytic activities of *h*Lon WT produced in both an *E. coli* Rosetta 2 (DE3) strain optimized for the expression of eukaryotic proteins and the *E. coli* C321 (DE3) strain designed for the incorporation of pCMF; we found no visible differences in their enzymatic activities (Supplementary Fig. S8).

**Figure 3:**
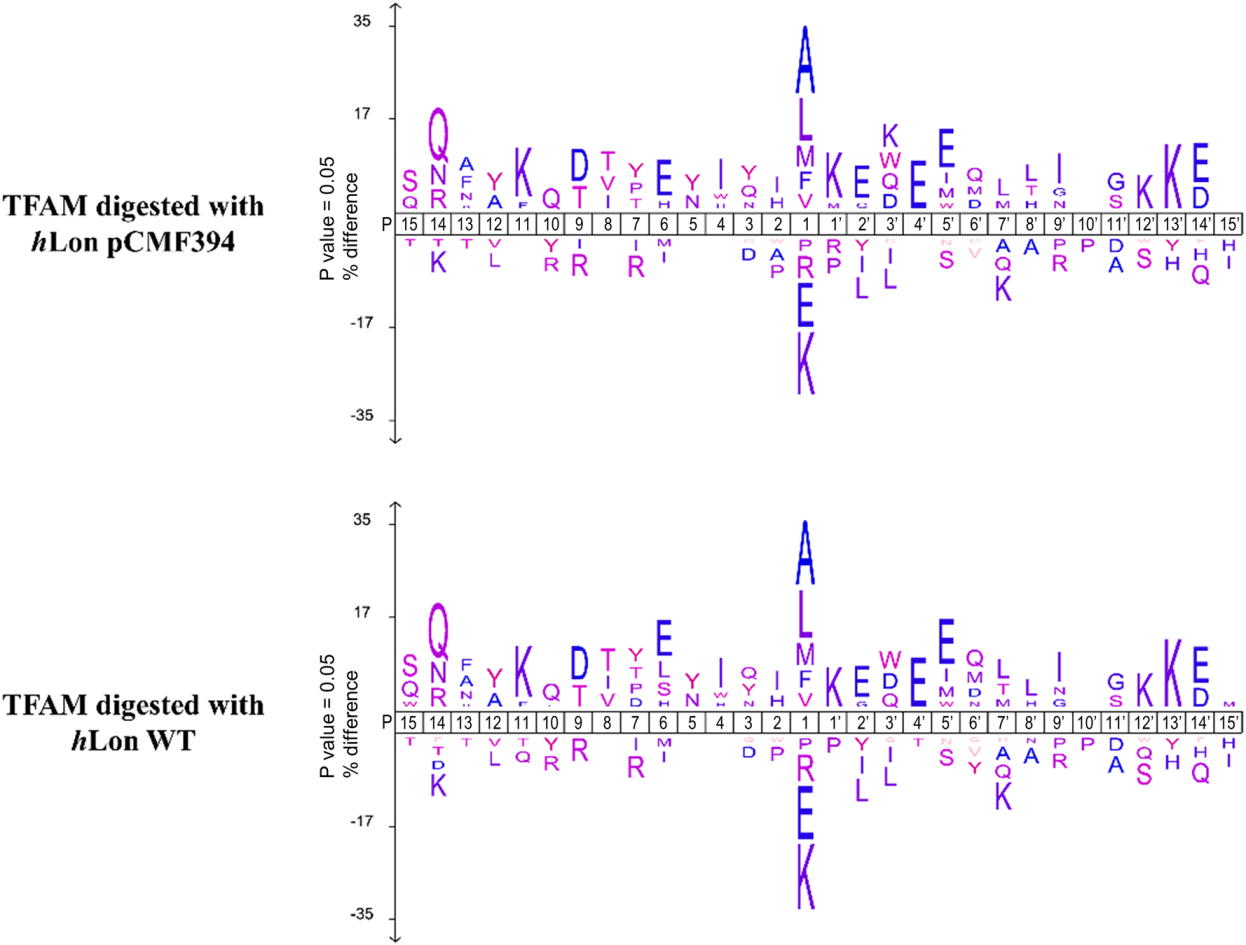
Visualization of the cleavage specificity of *h*Lon variants using TFAM as a substrate. TFAM peptides after *h*Lon cleavage were determined by mass spectrometry and conserved patterns of cleavage sites were visualized using iceLogo v.1.3.8. The positive set comprises the N-terminal and C-terminal cleavage windows (from − 15 to 15) of peptides with Andromeda score greater than 100 identified by MS/MS in at least one biological replicate of TFAM samples digested with *h*Lon_pCMF394 and *h*Lon WT. Cleavage windows (from − 15 to 15) of all theoretically possible TFAM cleavages were used as the negative set. The percentage difference in frequency for an amino acid in the positive and negative sets is given as the height of a letter in the amino acid stack. Only significantly over- and under-represented amino acids (threshold value *p* = 0.05) are shown.

In addition to tracking the change in the rate of proteolysis caused by the pCMF substitutions, we also examined the cleavage products produced by the *h*Lon digestion of TFAM to see if any changes in target sequences occurred. A mass spectrometry analysis of the reaction mixtures from *h*Lon WT and its pCMF394 phosphorylated version (in 2 biological replicates) revealed similar patterns of TFAM degradation products. In total 254 unique TFAM peptides (achieving a MaxQuant Andromeda score for the associated MS/MS spectra > 100) were identified by LC–MS/MS; 231 of these were produced by both *h*Lon versions. Both *h*Lon proteases created peptides of similar length and showed identical amino-acid cleavage specificity at position P1 (Fig. 3, Supplementary Fig. S9, Table S5) suggesting that phosphorylation at Tyr394 most likely does not influence the specificity of *h*Lon digestion but rather the overall rate of digestion.

The protease activities of the pCMF *h*Lon variants were quantified by measuring the fluorescence released by the digestion of fluorescein-isothiocyanate-labeled casein (FITC-casein). The results were consistent with the courses of the in vitro β-casein digestions on SDS-PAGE (Fig. 2). The fluorescence generated by digestion with *h*Lon WT after 30 min was more than three times higher than that measured for the pCMF186 version, while the pCMF394 variant produced almost no fluorescence signal at all (Table 1 and Supplementary Fig. S10). Moreover, we determined two additional *h*Lon enzymatic activities - its ATPase activity and its ability to degrade a fluorogenic peptide - glutaryl-Ala-Ala-Phe-MNA (peptidase activity). The basal ATPase activity of both *h*Lon pCMF variants, measured as the amount of inorganic phosphate released over time^30^ was visibly lower than that for *h*Lon WT; the stimulated ATPase activity of *h*Lon_pCMF186 measured in the presence of β-casein reached only about half the level of the wild-type stimulation. Whereas both basal and stimulated ATPase activities were almost undetectable for the pCMF394 mutant (Table 1 and Supplementary Fig. S11). The same pattern was observed by measuring the peptidase activities of pCMF186 and pCMF394. The pCMF186 variant had much lower basal and stimulated peptidase activities than *h*Lon WT, while the pCMF394 variant exhibited almost no peptidase activities (Table 2 and Supplementary Fig. S12).

**Table 1:** ATPase and protease activities for phosphorylated and phosphorylation-mimicking *h*Lon mutants.

Protein	ATPase activity (nmol·min^−1^)					Protease activity (RFU·min^−1^)	
	Basal	Stimulated	Stimulation	Frac. Basal	Frac. Stimulated	Rate	% WT
WT	0.137 ± 0.003	0.330 ± 0.010	2.409	1.000	1.000	46,400 ± 400	100
Y186E	0.105 ± 0.002	0.161 ± 0.004	1.533	0.766	0.488	27,600 ± 400	59.5
Y186F	0.136 ± 0.003	0.340 ± 0.010	2.500	0.993	1.030	49,600 ± 700	106.9
pCMF186	0.060 ± 0.001	0.101 ± 0.003	1.683	0.438	0.306	13,600 ± 300	29.3
Y394E	0.069 ± 0.001	0.160 ± 0.004	2.319	0.504	0.485	31,400 ± 600	67.7
Y394F	0.048 ± 0.001	0.262 ± 0.005	5.458	0.350	0.794	24,400 ± 200	52.6
pCMF394	0.005 ± 0.001	0.006 ± 0.001	1.200	0.036	0.018	1290 ± 90	2.8

Values are ± 1 standard error. nmol∙min^−1^ - nanomoles of PO_4_^3−^ released by *h*Lon per minute; Frac. Basal - ratio of the basal activity of the mutant to basal activity of the WT; Frac. Stimulated - ratio of the stimulated activity of the mutant to stimulated activity of the WT; RFU - relative fluorescence units; % WT - percent wild-type.

**Table 2:** Peptidase activity for phosphorylated and phosphorylation-mimicking *h*Lon mutants.

Protein	Peptidase activity (pmol·min^−1^)				
	Basal	Stimulated	Stimulation	Frac. Basal	Frac. Stimulated
WT	38.1 ± 0.3	71 ± 1	1.863	1.000	1.000
Y186E	28.7 ± 0.5	46.8 ± 0.5	1.631	0.753	0.659
Y186F	42.6 ± 0.2	97 ± 2	2.277	1.118	1.366
pCMF186	10.0 ± 0.2	18.0 ± 0.2	1.800	0.263	0.254
Y394E	17.3 ± 0.6	37.2 ± 0.6	2.150	0.454	0.524
Y394F	25.0 ± 0.2	59.2 ± 0.9	2.368	0.656	0.834
pCMF394	0.018 ± 0.003	0.150 ± 0.02	8.333	0.001	0.002

Values are ± 1 standard error. pmol∙min^−1^- picomoles of MNA released by *h*Lon per minute. Frac. Basal-ratio of the basal activity of the mutant to basal activity of the WT; Frac. Stimulated-ratio of the stimulated activity of the mutant to stimulated activity of the WT.

To more precisely determine the rate of ATP consumption, we quintupled the amount of enzyme used in the reaction. Under these conditions, pCMF394 did show a small degree of ATPase activity, but stimulation by β-casein was still not observed (Supplementary Table S6).

In contrast to the pCMF variants, the phosphorylation-mimicking *h*Lon mutants prepared by amino-acid exchange at least partly retained their caseinolytic activity. When either, Tyr186 or Tyr394, were mutated to phenylalanines (Y186F; Y394F), the caseinolytic ability of the resulting proteins substantially increased, with almost all β-casein digested within the first 5 min (Fig. 2). When Tyr186 and Tyr394 were exchanged for glutamates (Y186E; Y394E), the digestion of β-casein was visibly slower, particularly for Y394E, where almost one third of the protein substrate still remained after 15 min (Fig. 2B). The same approach was also used to study TFAM and the most significant digestion delay was observed in the reaction with Y186E, where more than half of the protein substrate remained undigested (Fig. 2C and Supplementary Fig. S7). Compared to TFAM digested by *h*Lon WT and Y186F serving as a control, the delay caused by introducing a glutamate instead of a tyrosine at Tyr186 site suggested a considerable change in *h*Lon functioning. The phospho-mimicking Y394E variant showed a slight decrease in TFAM digestion when compared to the wild-type or the Y394F control (Fig. 2C and Supplementary Fig. S7).

In nearly all studied *h*Lon variants prepared by amino-acid exchange, the FITC-casein digestion measurements agreed with the SDS-PAGE results. The highest FITC-caseinolytic activity was measured for the Y186F mutant, which exhibited the most rapid β-casein digestion and relatively fast TFAM degradation. The Y186E and Y394E had both a slower rate of substrate digestion and a significantly lower proteolytic activity compared to the wild-type. Surprisingly, the Y394F control had the lowest FITC-casein digestion even though it had the fastest digestion of both β-casein and TFAM (Table 1, Supplementary Fig. S10). The peptidase activities of these mutants followed the same pattern. The glutamate-containing mutants (Y186E; Y394E) again showed a lower basal ability to cleave the glutaryl-Ala-Ala-Phe-MNA peptide than the wild-type, though the peptidase activities observed in the presence of β-casein showed the same or even slightly higher stimulation levels than the wild-type (Table 2, Supplementary Fig. S12). The phenylalanine mutants (Y186F; Y394F) both showed relatively high basal peptidase activities and the highest stimulation rates, in agreement with their fast substrate degradation on gels.

The ATPase activities of Y186E and Y394E versions were also negatively affected. Both basal and stimulated ATPase activities were lower than that for *h*Lon WT. In fact, the stimulated activities of both barely reached the level of the basal ATPase activity of the wild-type (Table 1 and Supplementary Fig. S11). In contrast, both control mutants (Y186F; Y394F) exerted ATPase activities comparable to the standard *h*Lon. The Y394F mutant’s basal and stimulated levels were lower, however the stimulation rate in the presence of β-casein measured for Y394F was almost the same as for the wild-type (Table 1).

### *h*Lon—substrate complex formation

**Figure 4:**
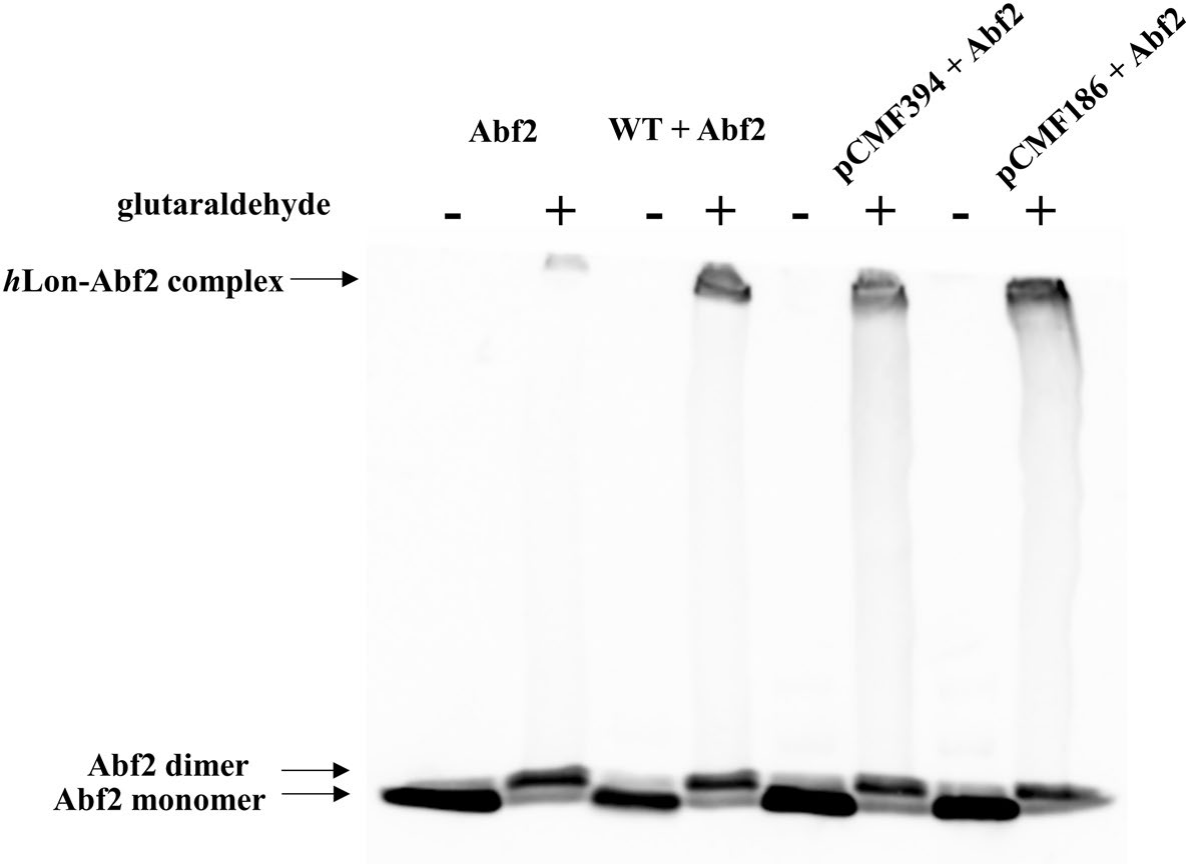
*h*Lon substrate-binding assay. A given amount of *h*Lon and Abf2 (in a 1:1.6 mass ratio) was crosslinked with 0.1% (v/v) glutaraldehyde for 40 min at room temperature in the presence of 2 mM ATP and 10 mM MgCl_2_ followed by separation on a 5% SDS-PAGE gel. The *h*Lon–Abf2 complex was visualized by immunoblot detection using an anti-Abf2 antibody. The samples incubated in the presence of a cross-linker are indicated by + . WT, wild-type *h*Lon; pCMF186, pCMF394 - phospho-mimicking *h*Lon mutants.

**Figure 5:**
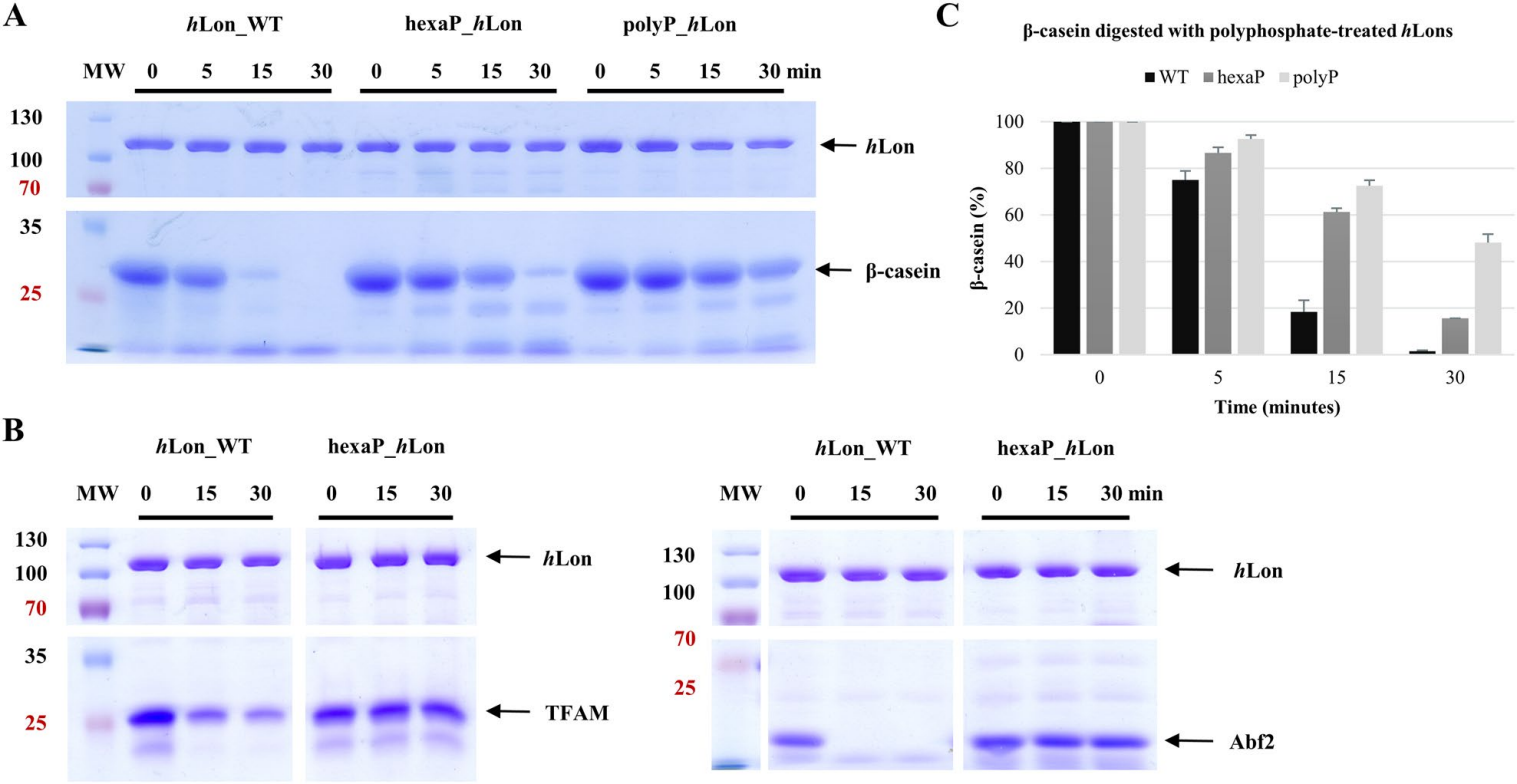
Protein substrates digested by hexa- and polyphosphate-treated *h*Lon. 5 μg β-casein, TFAM and Abf2 were incubated with 1 μg hexaP- or polyP-treated *h*Lon in reactions containing 2 mM ATP and 10 mM MgCl_2_. β-casein (**A**), TFAM and Abf2 (**C**) were digested by hexaP- and polyP-treated *h*Lon variants. The samples were withdrawn at the indicated times and loaded on a 12% SDS–polyacrylamide gels (only cropped images are shown here). MW, molecular weight marker; *h*Lon_WT, wild-type *h*Lon; hexaP_*h*Lon, hexaphosphate-treated *h*Lon; polyP-*h*Lon, polyphosphate-treated *h*Lon. Band intensity evaluation (**B**) of β-casein digestion shown in (**A**) was performed with ImageJ (v.1.53)^29^ separately for each studied protein. The height of a given bar represents the mean value, and the error bars show ± one standard deviation from at least three separate measurements.

Given the behavior of the pCMF394 variant we tested whether it is able to bind its protein substrate. *h*Lon WT and its two pCMF variants were incubated with Abf2, a *Saccharomyces cerevisiae* TFAM homologue, in the presence of a crosslinker. Our previous work^28^ showed that Abf2 can be used as an in vitro* h*Lon substrate, which is degraded rapidly in the first 15 min of the reaction (Fig. 5C). An Abf2 immunoblot assay of the cross-linked samples showed that an *h*Lon-Abf2 complex formed in all three cases (WT, pCMF186 and pCMF394) when glutaraldehyde was present (Fig. 4). In samples without a crosslinker (indicated by -), only bands corresponding to the Abf2 monomer were visible.

### Characterization of polyphosphate-treated *h*Lon

In mammals, polyphosphate was shown to accumulate within mitochondria. Under normal conditions, the levels of polyphosphate reach only micromolar concentrations^31^, however it increases rapidly in response to stress. Therefore, rather unsurprisingly, polyP was also shown to be highly abundant in several types of tumors^23^. To study the effect of polyP on the human Lon protease, we incubated *h*Lon WT with a polyphosphate (polyP) composed of 45 ± 5 phosphate residues and hexaphosphate (hexaP) composed of six phosphates; unbound polyphosphate was removed using size-exclusion chromatography. Both glutaraldehyde cross-linking (Supplementary Fig. S13) and SEC analyses (Supplementary Fig. S14A,B) showed that hexaP- and polyP-treated *h*Lons are still able to form functional oligomers. SEC also showed that both hexaP- and polyP-treated *h*Lon oligomers eluted in fractions shifted to higher molecular masses compared to the wild-type. This may indicate that hexaP or polyP directly interacts with *h*Lon, thereby slightly enlarging its hydrodynamic profile and increasing the negative charge on its surface. Besides, SEC–MALLS analysis separated two forms of *h*Lon: the first peak (eluting in the 13th ml) corresponded to an oligomer with a molecular weight close to that of a typical *h*Lon hexamer (~ 600 kDa) while the second peak (16th ml) was an *h*Lon monomer (~ 94 kDa) (Supplementary Fig. S14C).

The fact that hexaP and polyP remain bound to *h*Lon is supported by the digestion of hexaP- and polyP-treated samples with Proteinase K. In DAPI-stained native gels, a visible shift of the bands corresponding to polyphosphates between the Proteinase K-digested and Proteinase K-undigested *h*Lon samples occurred. The actual migration of the oligomeric hexaP- and polyP-treated *h*Lons is shown in the CBB-stained gels in Supplementary Fig. S15.

The thermal stabilities of hexaP- and polyP-treated *h*Lon samples were again determined by nanoDSF and compared to the wild-type protease (Supplementary Fig. S16 and Table S7). The denaturation temperatures of both hexaP- and polyP-treated *h*Lon forms were lower (by 3.2 ± 0.6 °C) than that of the untreated wild-type. Once again, the differences did not notably affect the protease variants themselves. Indeed, the aggregation onset temperatures of both hexaP- and polyP-treated samples were notably lower compared to the wild-type (Supplementary Table S7) suggesting that the polyphosphates destabilized the *h*Lon hexamer and thus increased its aggregation rate. Although these studies all indicate that hexaP and polyP bound to *h*Lon, they do not guarantee that the hexaP/polyP occupancy or stoichiometry were uniform in our hexaP- and polyP-treated *h*Lon samples.

### The influence of polyphosphates on *h*Lon activities

Interestingly, the in vitro caseinolytic assay showed that although both hexaP- and polyP-treated *h*Lon forms were able to form oligomers, their ability to digest β-casein was largely impaired. HexaP *h*Lon had barely half the caseinolytic activity the *h*Lon WT (about 84% of the β-casein had been digested after a 30-min reaction with hexaP *h*Lon, while roughly the same level of β-casein had been consumed after 15 min by the wild-type). The impairment was even more significant in the polyP-treated sample, where almost 50% of the β-casein remained untouched after 30 min (Fig. 5A,B). The digestions of TFAM and Abf2 were also considerably slowed (Fig. 5C).

These results were also supported by ATPase activity measurements. Both the basal and β-casein-stimulated ATPase activities of hexaP *h*Lon were only approximately half the levels of the wild type, while the ATPase activities of polyP *h*Lon reached only a fifth of the basal and β-casein-stimulated ATPase activities of *h*Lon WT (Supplementary Fig. S17, Table S8).

### Cryo-electron microscopy studies of *h*Lon

The structural effects of phosphorylation on *h*Lon were studied using cryo-EM. We focused on the ADP-bound forms because these modifications occur in Lon’s NTD, which is responsible for substrate binding. It is known that substrate binds to the inactive ADP-bound Lon and promotes ADP-ATP exchange. ATP is then hydrolyzed and the energy released is used to transport the substrate further into Lon’s proteolytic chamber, and to power the conformational changes accompanying Lon’s inactive-to-active state transformation^32^. Cryo-EM maps were obtained for Y186E, Y186F, pCMF186, Y394E, and Y394F with resolutions ranging from 2.9 to 8.5 Å (based on the 0.143 cut-off of the Fourier shell correlation curve) (see Supplementary Table S9 and Supplementary Figs. S18–S21). A structure of the pCMF394 variant could not be determined as the modification seems to prevent the formation of a stable hexameric head (Supplementary Fig. S22), and only transient forms where NTD could be identified in the 2D classes were observed. This is in contrast to previously reported wild-type *h*Lons and mutants, where the hexameric “head” is generally well structured and difficult to disrupt while the NTD is highly flexible and is more poorly resolved^15^. In the pCMF394 *h*Lon complex, the standard hexameric envelope of the “head” domain could be easily identified on the grids, however, it was so flexible that the alignment procedures could only have been performed with the NTD part alone. The head appears completely unable to form a stable conformation, which correlates with the activity loss observed above. We used the coordinates of the P1A state of *h*Lon WT protease reported by Mohammed et al*.*^16^ (PDB ID 7NFY) as an initial reference. In each case, the ATPase-protease “head” domain was substantially better resolved than the N-terminal “legs” domain. For the Y394E, Y186E, and Y394F structures, it was possible to build atomic coordinates into the “head” domain density, but only rigid body fit the 7NFY subdomains carried out for the Y186F and pCMF186 maps. The resolution obtained for the NTD domain densities was not sufficient to identify secondary structure elements in any of the studied maps, therefore, these parts of the cryo-EM densities were interpreted by rigid body fitting of the corresponding 7NFY region. ADP could be clearly resolved in the Y394E, Y186E, and Y394F structures. Unfortunately, residues 394 and 186 were not resolved in any of the structures. The structures do show that our substitutions in the “neck” do not affect the “head” domain, which agrees with the previous observations of the high flexibility of Lon’s NTD^15^. We should note here that in these studies, the Lon particles from all the *h*Lon variants we studied were very homogeneous. Apart from the intrinsic NTD flexibility, we did not see any additional sub-populations or intermediate “dynamic” states. This is in contrast to the situation observed in previous studies^15,16^. Overall, it appears that the mutant forms are less flexible than wild-type *h*Lon.

**Figure 6:**
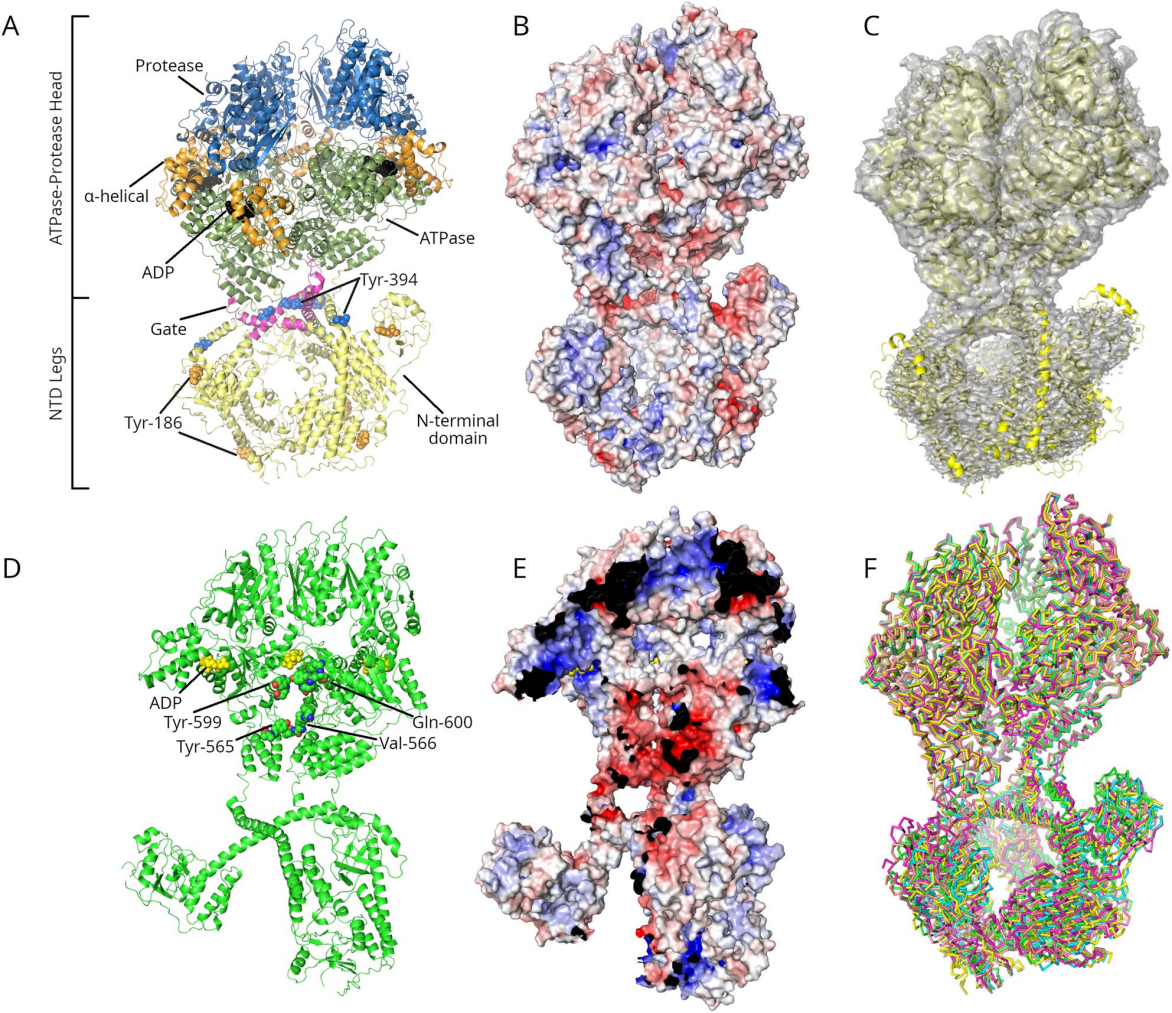
Cryo-EM structure of the Y394E *h*Lon mutant bound to ADP. All mutant structures are very similar, differing mostly in the location of the poorly ordered N-terminal domain; only the Y394E structure is therefore shown as it should be representative of the whole set. (**A**) The overall structure of the Y394E–ADP complex. The ATPase-Protease head is shown at top and the N-terminal domain (NTD) legs at bottom. The different subdomains are colored blue (protease domain), orange (α-helical domain), green (ATPase domain), and yellow (NTD); the triangular gate between the NTD and the entry to the ATPase-Protease domain is colored magenta. ADP is colored black; Tyr-186 and Tyr-394 are shown as van der Waals spheres. (**B**) The electrostatic surface of the exterior of *h*Lon. This surface and the one in panel (**E**) below are ramped at ± 10 kT/e. (**C**) The cryo-EM map of Y394E contoured at 4.0 RMSD. (**D**) A view of the interior of Y394E with the three chains closest to the cleft (**A**, **E**, and **F**) removed. ADP and residues Tyr-565, Val-566, Tyr-599, and Gln-600 are shown as van der Waals spheres. These residues were shown to be important for substrate translocation by previous studies^17^. (**E**) The same view as (**D**), showing electrostatic surface of the interior of the *h*Lon Y394E mutant (see also Supplementary Fig. S24). Overall, the NTD tends to be positive or neutral while the gate and the entry into the ATPase-Protease domain are strongly negatively charged. This is followed by a largely neutral chamber while the exit pore at the end of the proteolytic domain is strongly positively charged. The view in panels (**D**) and (**E**) is rotated by 30° with respect to those in the other panels in order to more clearly show the passage through the ATPase-Protease domain. See also Supplementary Fig. S25 where the ATPase-protease unit of a K398A *h*Lon mutant bound to a substrate peptide is shown. (**F**) Overlaps of the Y394E (green), Y394F (cyan), Y186E (magenta), Y186F (yellow), and pCMF186 (salmon) structures. It can be seen that the head domains overlap closely while the NTD legs show the greatest positional flexibility.

All structures were surprisingly very similar; consequently, only Y394E, the highest resolution one, is shown in Fig. 6 and used for comparison with previous structures. Cryo-EM density maps of all structures may be found in Supplementary Fig. S23. Y394E is similar to the ADP-bound forms determined previously by Shin et al*.*^14^ (PDB ID 6V11), Mohammed et al.^16^ (7OXO) and Gesé et al.^17^ (7P0B) (root-mean-squared deviations of 1.83 Å, 1.810 Å, and 4.0 Å, respectively). Y394E forms the same lock-washer structure as these other structures and has roughly the same twist (50–55°) and rise (8.0 Å as measured by us for all structures) as the previously reported structures together with a gap around 23° wide between two of the subunits. Curiously, none of the previous studies closely examined the electrostatic characteristics of the *h*Lon structure. The outer electrostatic surface of *h*Lon is shown in Fig. 6B and the interior surface is shown in Fig. 6E. *h*Lon’s external surface has a mixture of charged and neutral areas with none of them being outstandingly large. The interior of *h*Lon, however, clearly has a polar character. At the neck region, where the NTD and ATPase domains come together, the protein is strongly negatively charged. This negative charge persists along the entire substrate translocation channel, including the sites of the Tyr565-Val566 and Tyr599-Gln600 residues in the pore loops which were shown to be involved in substrate translocation. At the end of the substrate translocation channel, the head opens out into a wide and relatively nonpolar cavity. On the far side of this cavity is the protease domain, which has a slightly negative charge in the area of the active site, but a strong positive charge lining the exit pore at the distal end of the ATPase-Protease domain. This pattern can also be seen in a substrate-bound K898A mutant structure (Supplementary Fig. S25).

## Discussion

Human mitochondrial Lon protease has been found to be phosphorylated on multiple sites. The phosphoproteomic studies currently reveal nine phosphorylated serines, five phosphorylated tyrosines and three phosphorylated threonines within *h*Lon with seven modified residues located in its N-terminal domain and six in its AAA^+^ domain. To date, Tyr394 in *h*Lon’s N-terminal substrate binding domain is the most frequently modified amino acid, identified by more than 50 proteomic analyses performed on disease tissues originating in pancreatic carcinomas, colorectal cancer, lung cancer, atrial fibrillation, and ventricular tachycardia^19^.

Recently, mitochondrial *h*Lon was shown to be phosphorylated by the Akt1 kinase, which accumulates in mitochondria under the stresses (e.g., hypoxia) that contribute to malignant growth in prostate cancer^9,22^. Akt1 phosphorylates *h*Lon at Ser173 and Ser181, both of which enhance its proteolytic activity^9^. Both sites reside relatively close to each other in the N-terminal substrate-binding domain, which is generally considered the most flexible and the most variable part of Lon.

Here, we studied the effects of phosphorylating the only two of these tyrosines (Tyr186 and Tyr394) located in *h*Lon’s NTD (Tyr186 lies very close to the previously studied serines, Ser173 and Ser181) on the structure, stability, and activities of *h*Lon. We prepared three modified forms of each residue. First, we replaced each tyrosine with glutamate, which has the effect of introducing a negative charge to this site. However, phosphotyrosine is both bulkier and substantially less conformationally labile than glutamate; consequently, we also substituted the phosphotyrosine-mimicking non-natural amino-acid pCMF at each location. This both introduces a negative charge and increases the bulk of the residue. Finally, we mutated tyrosine to phenylalanine at each site to verify that the loss of the reactive capability imparted by the tyrosine hydroxyl group was not primarily responsible for the effects. We then evaluated the effects of these six substitutions on the structure, stability, ATPase, protease, and peptidase activities of *h*Lon.

All prepared *h*Lon variants retained their oligomeric states (Supplementary Fig. S4) and thermal stabilities up to at least 48 °C and above (Supplementary Table S4). The Y186F and Y394F control mutations had no significant effect on the ability of *h*Lon to degrade either β-casein or TFAM. Quantifying the protease activity with FITC-casein showed that the Y394F mutant had only about 50% of the wild-type activity. Similarly, the basal ATPase and peptidase activities of Y394F were lower than those of *h*Lon WT. However, the effect of substrate binding had the greatest stimulatory effect on the ATPase and peptidase activities of Y394F. Altogether, this suggests that the Y186F mutation is largely neutral, while the Y394F mutation has some downstream effect on the ATPase and peptidase activities, which is largely corrected by the binding of a substrate.

The replacement of Tyr186 and Tyr394 with the negatively charged glutamate diminishes all *h*Lon activities, while replacement with pCMF either greatly reduces or abolishes them. In particular, Y186E is still able to degrade β-casein, but more slowly, while it loses the ability to degrade TFAM. Y394E, by contrast, still digests both. Quantifying the protease activity using FITC-casein shows that both proteins retain at least 60% of the wild-type activity. Similarly, the ATPase and peptidase activities of these two proteins in the presence of β-casein are between 50 and 80% of the wild-type values. The stimulation of the ATPase and peptidase activities for Y186E has been notably reduced to only 1.5–1.6 times the basal activity, while that of Y394E is still of the wild-type level, although its basal activity is lower. Altogether, it appears that both mutations perturb the activities of *h*Lon. Given that the Y186E mutation affected the ability of *h*Lon to bind at least one of its substrates, they are likely to do this via different mechanisms.

Finally, the pCMF substitutions greatly inhibit TFAM digestion without affecting the binding of a substrate or the cleavage specificity, reduce β-casein digestion, and greatly reduce (pCMF186) or effectively abolish (pCMF394) FITC-casein digestion. pCMF186 has very low ATPase activity and peptidase activity, and the activities of pCMF394 are near the detection limit. Overall, pCMF186 is similar to Y186E, but more extreme, while pCMF394 is qualitatively different from Y394E.

All of the substitutions that introduced a negative charge, Y186E, pCMF186, Y394E, and pCMF394, decreased the melting temperature of *h*Lon by at least 2 °C while the Y186F and Y394F mutations had the same thermal stability as *h*Lon WT. Structurally, only the pCMF394 substitution had a large impact: the cryo-EM structures of the ADP-bound forms of the Y186E, Y186F, pCMF186, Y394E and Y394F mutants were all very similar, while the pCMF394 substitution destabilized the formation of the ATPase-protease hexamer (Supplementary Fig. S22). Because the ATPase and protease activities of Lon are known to depend upon the proper formation of the Lon hexamer, its disruption by this replacement could account for this mutant’s lack of activity.

The lower *h*Lon activities caused by phosphorylation may suggest similar effects as were previously observed by silencing the mitochondrial *h*Lon in RKO colon cancer cells^33^. Lon-silenced cells displayed altered expression of mitochondrial proteins, lower level of mtDNA transcription, reduced oxygen consumption and ATP synthesis, leading to fragmented mitochondria and, eventually, to cell death. Similar observations were also made in *S. cerevisiae* Lon/Pim1 knock-down mutants^34^ and lymphoma cells treated with CDDO, a synthetic triterpenoidal inhibitor of Lon^35^.

Similarly to the other recently-determined cryo-EM *h*Lon structures, the N-terminal domain was resolved at low resolution which limits a more accurate interpretation. However, we could have some confidence in the positions of Tyr394 and Tyr186. First, the three recently reported cryo-EM structures of *h*Lon all modelled the N-terminal domain independently and all placed the residues in the same places (one of these^16^ used a total of three different methodologies to verify the overall fold). Second, an AlphaFold model constructed using the NTD of the *h*Lon sequence agrees with the location of these residues. Finally, Gesé et al*.*^17^ prepared and characterized a Y394A point mutant and found that both TFAM degradation and ATP hydrolysis were greatly reduced, which supports the placement of this residue in the triangular gate region.

**Figure 7:**
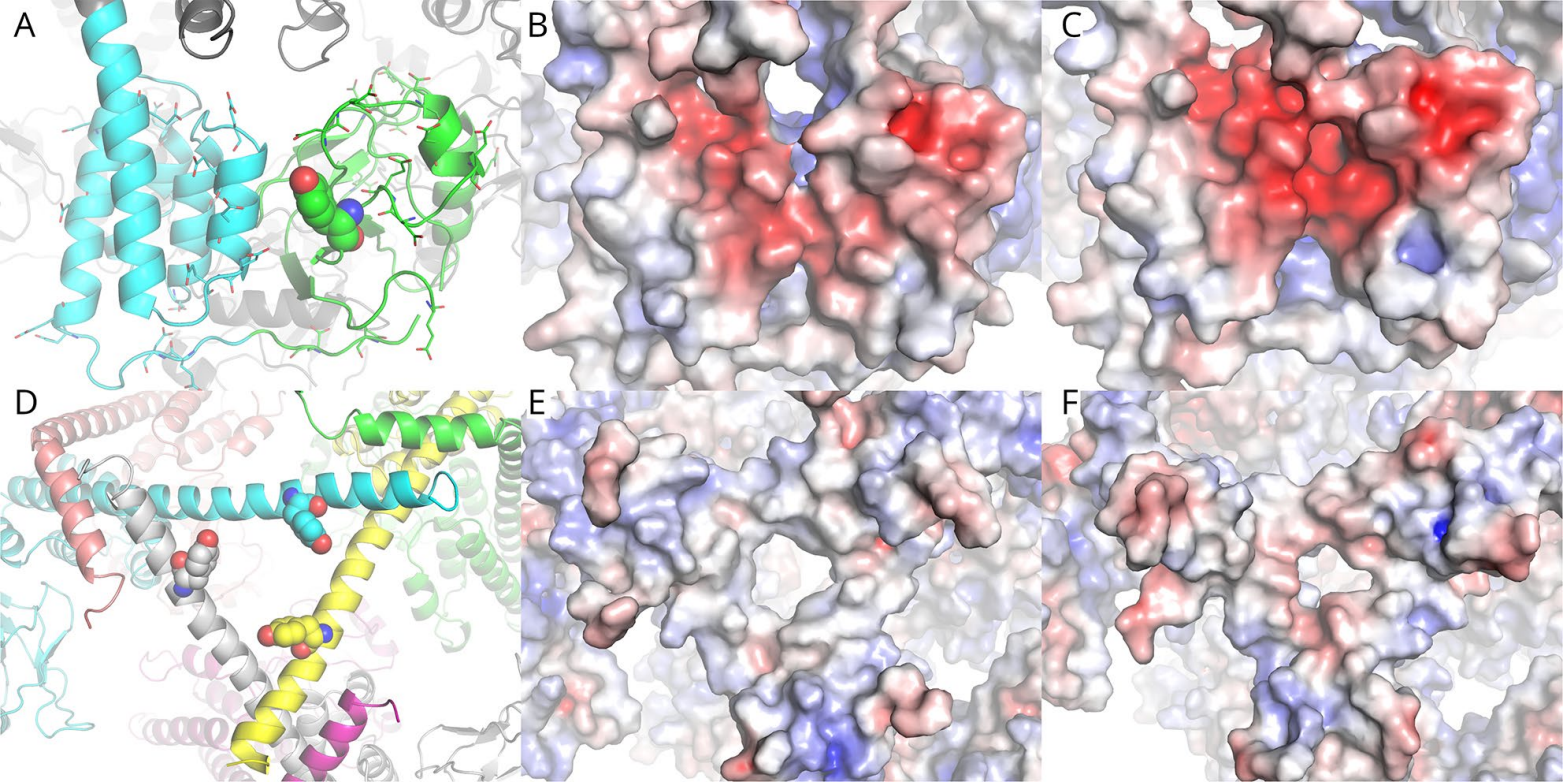
Effect of Tyr → Glu substitutions on the *h*Lon NTD electrostatic surface. (**A**) The NTD of *h*Lon consists of two lobes, an N-terminal β-sheet containing one (green) and an all α-helical one (cyan) followed by a long α-helix which is bent in every second chain. Tyr186 (spheres) is located at the junction between these subdomains and is surrounded by negatively charged Asp and Glu residues (sticks). The effect of these residues is to make the electrostatic surface of this area (**B**) strongly negatively charged. The strongest negative charge between the two domains is roughly − 7 kT/e while the average charge between the interfaces is roughly − 4 kT/e. (**C**) The Y186E substitution increases this negative charge. The average charge in the cleft is now around − 7 kT/e while the highest charge between the two domains is roughly − 12 kT/e. (**D**–**F**) The effect of the Y394E substitution on the electrostatic surface of the triangular gate separating the NTD and the ATPase-Protease domain. These surfaces are calculated over the NTD only: it was found that the strong negative charge around the opening of the ATPase-Protease domain had a dominant effect on the overall electrostatics of the gate and that changes in the relative orientation of the NTD and ATPase-Protease domain had a greater effect than the individual substitutions. (**D**) The NTD gate from *h*Lon WT structure (PDB ID 7OXO^16^); Tyr394 is shown as spheres. (**E**) The electrostatic surface of the wild-type and (**F**) the Y394E mutant. It can be seen that in the wild-type protein, the gate is neutral to positively charged, while in the Y394E mutant it has become neutral to negatively charged. All electrostatic surfaces are shown at ± 10 kT/e.

Given this, it should still be possible to infer some of the likely structural consequences of these substitutions. A number of crystal structures of the N-terminal domain from various bacterial Lon species have shown that the NTD consists of two subdomains, a β-sheet containing N-terminal and α-helical lobes connected by a flexible linker. The C-terminal end of this α-helical subdomain appears able to adopt a variety of conformations depending on the local environment. Although none of the cryo-EM structures of Lon to date have resolved the fold of the NTD, they do allow us to see that the shape of the *h*Lon NTD is consistent with those from bacteria. The two subdomains are packed loosely together (an analysis of the domain interface using PDBePISA^36^ gave the domain interface a significance score of 0.00) and Tyr186 can be found in the cleft between the two domains on the β-sheet domain containing side. Tzeng et al.^18^ found that unfolded substrates interacted with the β-stranded subdomain through two hydrophobic patches, however Tyr186 is found on the opposite side of the domain from these patches, so the drop in proteolytic digestion exhibited by the Y186E and pCMF186 substitutions is unlikely to be due to direct interference with substrate binding. Tyr186 is, however, surrounded by negatively charged glutamate and aspartate residues from both subdomains, putting it in the middle of a large, negatively-charged patch. Phosphorylation of Tyr186 would therefore place an additional negative charge on a large group in the middle of this cleft, which could force the subdomains apart, thereby interfering with the proper binding and recognition of Lon substrates (Fig. 7). This explanation nicely covers the effects of the Y186E and pCMF186 substitutions on Lon’s ability to degrade protein substrates: The introduction of a negative charge by Y186E halts TFAM digestion and diminishes β-casein and FITC-casein digestion. Tzeng et al*.*^18^ found that *Meiothermus taiwanensis* (*Mta*LonA) Lon is able to degrade the intrinsically unstructured protein α-casein without the NTD but that the NTD was necessary for native protein digestion. Kereϊche et al*.*^15^, however, found that *h*Lon lost the ability to digest β-casein when the first 270 residues, corresponding to the β-sheet-containing subdomain, were deleted. Thus, the way Lon interacts with natively unstructured proteins is different from the way that it deals with native proteins, which may account for the difference between β-casein and TFAM.

Because the region containing residues 370–407 adopts two different conformations, Tyr394 can be found in different locations on alternate chains. In the first conformation, Tyr394 appears near the break where the long α-helix is interrupted and bends. In this location, Tyr394 may interact with residues Lys368 and Glu371 from a neighbouring subunit. As there is enough space, the replacement by phosphotyrosine would unlikely cause any serious disruption of the interaction. This location is, however, fairly easy to access with a kinase. In the second conformation, Tyr394 lines the triangular gate joining the NTD to the ATPase-protease “head” domain. Phosphorylating this tyrosine would hinder or block this passage, thereby largely eliminating the Lon activity, even if the hexamer did remain intact. A Y394E substitution would not obstruct this channel but might change the dynamics of the Lon-substrate interaction, thereby slowing passage. One potential issue is that this site cannot be easily accessed by a kinase in most of the known configurations. If there is a large conformational change in the NTD (for example, like that observed for *Mta*LonA^18^) or if the conformations of the individual chains can exchange, then this site might become accessible. It is also possible that Tyr394 is phosphorylated in the cytosol before *h*Lon is transported into the mitochondria and, consequently, before it assumes its final oligomeric form. This would also explain the phosphorylation sites detected in *h*Lon’s MTS (Ser4, Thr5, Tyr7 and Thr33) identified by MS.

Lon protease was also found to bind polyphosphate, a polymer that is highly abundant in cancer cells, but present in only low levels in normal cells^23,37,38^. Boyineni et al.^23^ detected a large amount of polyP in several primary tumor types, including human bronchioloalveolar adenocarcinoma, invasive ductal adenocarcinoma, small intestine adenocarcinoma, prostate adenocarcinoma and medulloblastoma. Interestingly, large amounts of polyP were found extensively, but not exclusively, inside the mitochondria or in close proximity.

Bacteria also produce polyP in response to multiple stressors, including amino-acid starvation, oxidative stress, heat shock, salt stress, and heavy metal exposure^39–42^. In *E. coli*, the complex of polyP with Lon protease plays an important role in its adaptation to amino-acid starvation^24,43^. *Ec*Lon was shown to degrade 4 (L1, L3, L6 and L24) of the 9 large subunit ribosomal proteins in the presence but not in the absence of polyP. Moreover, polyP stimulated the degradation of purified L9, L13, and L17 only when the subunits were not assembled in ribosomal complexes^24^. *Ec*Lon-mediated degradation also depended on the length of the polyP chain: while *Ec*Lon bound to a polyP containing hundreds of phosphates (P700) was effective in degrading the free ribosomal proteins S2 and L13, *Ec*Lon was less stimulated when bound to a polyP with a shorter chain length (65 phosphate residues), and *Ec*Lon bound to a 15-residue polyP was largely inactive. On the other hand, *Ec*Lon-mediated degradation is not always simulated by polyP. For example, the degradation of (MBP)-SulA, an *E. coli* cell division inhibitor, by *Ec*Lon was slightly inhibited when bound to polyP^24^.

In humans, the level of polyP is low under normal conditions but significantly increases in cancers that, in some aspects, resemble starvation. We have shown that human Lon is able to form stable complexes with polyP, but that the effect is significantly different from that in *E. coli*. When polyP binds to *h*Lon, it substantially reduces its degradation of β-casein and the endogenous substrates, TFAM and Abf2. This effect was more pronounced with polyP-treated *h*Lon than with hexaP-treated *h*Lon. These results are also supported by ATPase activity measurements showing that both polyphosphate-treated *h*Lons reached barely half the level of the wild-type with both basal and stimulated activities. Interestingly, the β-casein stimulation of both polyphosphate-treated *h*Lons was comparable to the wild-type (approx. twofold). It seems that protein degradation in the presence of polyP is a highly complex process and depends on the nature of degraded proteins, the length of the polyP-chain, the amount of polyP bound to Lon, and also the possibility of the substrate forming polyP-complexes of its own.

Several recent studies have highlighted the role of mitochondria in cancer cells, not only as powerhouses but also as dynamic signaling organelles controlling cell survival and death, motility, and resistance to treatment^44–46^. Lon is one of the crucial mitochondrial proteins involved in the very precise regulation of essential mitochondrial functions, and several of its PTMs are tightly connected to cancer and metastases. Our results show that one of those PTMs, the phosphorylation of two residues in *h*Lon’s NTD, has the effect of greatly reducing Lon’s activities without having any effect on its overall hexameric conformation. We also find that the binding of *h*Lon to polyphosphate chains of different lengths has similar effects and that a longer chain seems to be more inhibiting.

In general, lower *h*Lon activities^34–36^ can significantly affect overall mitochondrial homeostasis. More specifically, it can lead to a decrease in mitochondrial ATP synthesis, resulting in a metabolic transition from oxidative phosphorylation to glycolysis, as observed in cancer cells. Both phosphorylation and polyphosphate binding might therefore enable Lon to rapidly convert between a non-functional and fully functional state, thereby representing a very effective system by which mitochondria could contribute to tumor cell growth through their biosynthetic capabilities rather than their energy producing ones. Further study of the effects of phosphorylation on other phosphorylation sites identified in *h*Lon could provide a more complex picture of *h*Lon’s role in the mitochondrial dynamics occurring in cancer progression.

## Methods

*Microbial strains and growth media: Escherichia coli* DH5α (F^–^ φ80*lac*ZΔM15 Δ(*lac*ZYA-*arg*F)U169 *rec*A1 *end*A1 *hsd*R17(r_K_^–^, m_K_^+^) *pho*A *sup*E44 λ^–^*thi*-1 *gyr*A96 *rel*A1) (NEB) was used to amplify plasmid constructs. *E. coli* Rosetta 2 (DE3) cells (F^–^*omp*T *hsd*S_B_ (r_B_^–^, m_B_^–^) *gal dcm* (DE3), pRARE2 (Cam^R^)) (Novagen) and *E. coli* C321 (DE3) cells (MG1655 Δ(*ybh*B-*bio*AB)::zeo^R^ Δ*prf*A; all 321 UAG codons replaced with UAA; (DE3))^26^ were used to produce recombinant *h*Lon versions and phospho-mimicking *h*Lon mutants, respectively. *E. coli* DH5α cultures were grown at 37 °C in LB medium (1% (w/v) bactotryptone, 0.5% (w/v) yeast extract, 1% (w/v) NaCl, pH 7.5) containing 100 μg/ml ampicillin. *E. coli* Rosetta 2 (DE3) cultures were grown at 37 °C in TB medium (1.2% (w/v) bactotryptone, 2.4% (w/v) yeast extract, 72 mM K_2_HPO_4_, 17 mM KH_2_PO_4_, 0.4% (w/v) glycerol) containing 0.5% (v/v) glucose with 0.5 mM IPTG, 100 μg/ml ampicillin and 34 μg/ml chloramphenicol. *E. coli* C321 (DE3) cultures transformed with pEVOL plasmid^27,47^ producing the aminoacyl tRNA and aminoacyl-tRNA synthetase for pCMF (*para*-carboxy-methyl-phenylalanine) incorporation were first grown at 37 °C for 4 h in TB medium containing 1.5 mM pCMF and 0.2% (w/v) *L*-arabinose with 100 μg/ml ampicillin and 34 μg/ml chloramphenicol followed by a 20-h expression at 23 °C induced by 0.5 mM IPTG.

*Site-directed mutagenesis of hLon:* The incorporation of an *amber* stop codon (TAG) at desired positions within *h*Lon (Tyr186 and Tyr394) was achieved using QuikChange Site-directed Mutagenesis Kit (Agilent Technologies) according to the manufacturer’s instructions. The same protocol was followed in replacement of *h*Lon’s Tyr186 and Tyr394 to Glu186, Phe186, Glu394, and Phe394. DNA oligonucleotides (Supplementary Table S1) were purchased from Sigma Genosys. The sequence of each construct was verified by DNA sequencing in Macrogen.

### Expression and purification of recombinant proteins

*Expression and purification of a wild-type human Lon and phosphotyrosine-mimicking human Lon variants:* The wild-type human Lon was expressed and purified as described in Ambro et al*.*^48^. The phosphotyrosine-mimicking human Lon variants (Supplementary Table S2) were expressed in *E. coli* Rosetta 2 (DE3) or *E. coli* C321 (DE3) as stated above. Cells were harvested, resuspended in solubilization buffer A (40 mM HEPES, pH 8.0; 150 mM NaCl; 10 mM MgCl_2_; 20% (v/v) glycerol) and sonicated on ice. The cell lysate was centrifuged for 30 min at 100,000 × g and the supernatant was loaded onto a NiNTA Agarose column (Qiagen). The column was washed with five column volumes of buffer A containing 40 mM imidazole. The protein fraction bound to the column was then eluted in buffer A with a stepwise gradient of imidazole (0.1—0.5 M). Elutions containing *h*Lon mutants were pooled, concentrated and loaded onto a Superose 6 10/300 GL column (Cytiva) in buffer A with 5% (v/v) glycerol. The incorporation of pCMF in *h*Lon_pCMF186 and *h*Lon_pCMF394 was verified by the MS–MS analyses.

*Preparation of hexaphosphate- and polyphosphate-treated hLon versions:* After size-exclusion chromatography (SEC) on a Superose 6 10/300 GL column (Cytiva) the fractions containing wild-type *h*Lon hexamer were pooled and concentrated to a smaller volume. The sample was then incubated with 50 mM hexaphosphate (sodium hexametaphosphate) or polyphosphate (sodium phosphate glass Type 45; Sigma S4379) in 20 mM HEPES, pH 8.0, 150 mM NaCl, 5% glycerol at 37 °C for 60 min and afterwards separated on the same SEC column. The fractions containing hexa- and polyphosphate-treated *h*Lon were pooled and concentrated for further use.

*Expression and purification of TFAM and Abf2:* Recombinant proteins, TFAM and Abf2, were expressed and purified as described in Kunova et al*.*^28^.

*Purity analysis and determination of protein concentration:* All purified proteins were concentrated on a Microsep Advance Centrifugal Device (10 K, and 30 K MW cut-offs) (Pall) and analyzed by SDS-PAGE and Coomassie Brilliant Blue R250 staining. The protein concentration was determined using the Pierce bicinchoninic acid assay (BCA).

*SEC–MALLS:* MALLS measurements were performed with an Agilent Technologies 1260 Infinity II, HPLC system equipped with a miniDAWN^®^ TREOS^®^ II light-scattering detector (Wyatt Technology Corporation) and a Shodex (RI-501) refractive index detector. A Superose 6 Increase 10/300 GL column (Cytiva) was equilibrated in 40 mM HEPES, pH 8.0; 150 mM NaCl; 5% (v/v) glycerol, and a 100 μl of purified *h*Lon WT and a hexaP-treated *h*Lon at a concentration of 6.4 mg/ml were loaded onto the column in the same buffer. The data were analyzed using ASTRA software (Wyatt Technology Corporation). 100 μl of BSA (2 mg/ml) were used as a reference.

*Chemical cross-linking of hLon:* Cross-linking of *h*Lon variants was performed as previously described in van Dijl et al*.*^49^. Briefly, 3 µg of protein was cross-linked with 0.1% (v/v) glutaraldehyde for 60 min at laboratory temperature in 50 mM HEPES, pH 8.0, 10 mM MgCl_2_, 2 mM ATP and then visualized on a 5% SDS-PAGE gel stained with Coomassie Brilliant Blue R250.

*Immunoblot analysis:* Proteins separated by a glycine-buffered SDS-PAGE were transferred from polyacrylamide gel to a nitrocellulose membrane (GE Healthcare) using a semi-dry transfer method. The proteins on the membrane were detected by primary antibodies (rabbit IgG fraction) directed against yeast Abf2 (BioGenes). Membrane-bound primary antibodies were then detected using peroxidase-conjugated secondary antibodies (anti-rabbit IgG, Jackson ImmunoResearch), incubated with chemiluminescence detection reagents and visualized by Azure 200 (Azure Biosystems).

*hLon-mediated digestion of substrates:* The given amounts of substrate proteins were incubated with *h*Lon versions in a reaction buffer (50 mM Tris–HCl, pH 8.5, 10 mM MgCl_2_, 2 mM ATP). The reactions were carried out at 37 °C for 30 min with aliquots withdrawn at the indicated times and the products were analyzed by SDS-PAGE and Coomassie Brilliant Blue R250 staining.

### ATPase, peptidase and protease activities of *h*Lon

ATPase, peptidase and protease assays were performed as described in Ambro et al*.*^48^. In brief, to measure the ATPase activity, 5 µg (or 25 μg when needed) of *h*Lon was incubated at 37 °C in 50 mM Tris–HCl pH 8.0, 40 mM MgCl_2_, 0.5 mM ATP. Aliquots were withdrawn every minute from 0 to 11 min and absorbance at 660 nm was measured. Activity stimulation by the substrate was determined by assaying the ATPase reaction in the presence of 25 µg β-casein. To estimate the peptidase activity, 5 µg of *h*Lon was incubated at 37 °C in 50 mM Tris–HCl pH 8.0, 40 mM MgCl_2_, 0.5 mM ATP with 0.25 mM of the fluorogenic peptide glutaryl-Ala-Ala-Phe-MNA in the absence or presence of 25 µg β-casein. The fluorescence was measured (excitation wavelength 340 nm, emission wavelength 440 nm) every 50 s for 20 min in a black 96-well plate (Thermo Fisher Scientific) in Synergy H1 plate reader (BioTek). To measure the protease activity, 7.5 µg of FITC-casein were cleaved by 5 µg of *h*Lon version in 50 mM Tris–HCl pH 8.0, 40 mM MgCl_2_, 0.5 mM ATP at 37 °C. Measurements were taken every 30 s for 20 min at excitation wavelength 487 nm and emission wavelength 528 nm.

*Nano differential scanning fluorimetry (nanoDSF) measurement:* Thermal stability and aggregation of measured samples were determined by the nanoDSF instrument Prometheus NT.48 using PR.ThermControl v2.3.1 software (NanoTemper Technologies, Germany). A given amount of proteins was loaded onto a nanoDSF in standard grade capillaries. The thermal unfolding was measured using the intrinsic tryptophan and tyrosine fluorescence at emission wavelengths of 330 nm and 350 nm continuously in a thermal ramp from 20 to 95 °C with a heating rate of 1 °C/min.

### Proteomic analysis

The phosphorylation-mimicking pCMF *h*Lon versions were reduced with 5 mM dithiothreitol, alkylated with 15 mM iodoacetamide, digested with trypsin (1:25 w/w) overnight and purified by microtip C18 SPE. The reaction mixtures of TFAM digested with *h*Lon versions were directly purified by microtip C18 SPE. The separation of peptides by HPLC was performed in a Dionex UltiMate 3000 RSLCnano system (Thermo Fisher Scientific), the samples were loaded onto a trap column (PepMap100 C18, 300 μm × 5 mm, 5-μm particle size; Thermo Fisher Scientific) and separated in an EASY-Spray C18 analytical column with integrated nanospray emitter (PepMap RLSC C18, 75 μm × 500 mm, 2-μm particle size, Thermo Fisher Scientific) using a 60-min gradient (3–43% B). The two mobile phases used were: 0.1% formic acid (v/v) (A) and 80% ACN (v/v) with 0.1% formic acid (B). Eluted peptides were sprayed directly into an Orbitrap Elite mass spectrometer (Thermo Fisher Scientific), and spectral datasets were collected in a data-dependent mode using the Top15 strategy to select precursor ions for HCD fragmentation^50^. Each of the samples was analyzed in technical duplicates. The resulting datasets were processed by MaxQuant, version 1.6.17.0^51^, with the built-in Andromeda search engine. Carbamidomethylation (C) was set as a permanent modification and oxidation (M) and Tyr > pCMF were set as variable modifications. The searched database consisted of amino-acid sequences of recombinant *h*Lon and TFAM and *E. coli* reference proteome downloaded from Uniprot. Identified protein groups and pCMF sites for pCMF *h*Lon versions are listed in Supplementary Table S3. Identified peptides of TFAM digested with *h*Lon versions are listed in Supplementary Table S5.

*Visualization of hLon variants’ cleavage specificity:* The conserved patterns of *h*Lons’ cleavage sites in TFAM protein were visualized by iceLogo v.1.3.8 software^52^. The positive set comprised the N-terminal and C-terminal cleavage windows (from − 15 to 15) of TFAM peptides with score greater than 100 identified by MS/MS in at least one biological replicate of TFAM samples digested with *h*Lon pCMF394 and *h*Lon WT, respectively. Cleavage windows (from − 15 to 15) of all theoretically possible TFAM cleavages were used as the negative set (Supplementary Table S5).

### Cryo-electron microscopy (Cryo-EM) imaging

Three microliters of the sample (1.3 mg/ml) were applied to freshly glow-discharged transmission electron microscopy (TEM) grids (Protochips, Cu, 300 mesh, R1.2/1.3) and vitrified in liquid ethane using a Thermo Fisher Scientific (TFS) Vitrobot Mark IV (4 °C, 100% rel. humidity, 30 s waiting time, 3 s blotting time). The grids were mounted into Autogrid cartridges and loaded onto a Talos Arctica (TFS) transmission electron microscope (TEM) for screening prior to data acquisition. The Y394E *h*Lon data were collected using Titan Krios TEM (TFS) operated at 300 kV using SerialEM (https://bio3d.colorado.edu/SerialEM/)^53^. The microscope was aligned for fringe-free imaging. The data were collected on a K3 direct electron detection camera (Gatan) positioned behind a Gatan Imaging Filter at a calibrated pixel size of 0.834 Å/px (nominal magnification 105 k×). The energy selecting slit was set to 10 eV. The data from a 2.0 s exposure were saved into 40 frames containing an overall dose of 40 e/Å^2^. The Y394F, Y186E, Y186F, and Y186 pCMF *h*Lon datasets were all recorded in the same manner using Talos Arctica (TFS) equipped with a post-GIF K2 camera and operating at 200 kV. The final pixel sizes were 0.783 Å/px at the nominal magnification of 165 k×. The data from 5.0 s exposures were saved into 40 frames containing overall doses of 40 e/Å^2^ and the energy selecting slit was set to 20 eV in each case. The datasets comprised 1376 and 12,393 movies in total for Y394F and Y394E, respectively. The image underfocus varied between − 1 to − 2 μm for Y394E, − 2 to − 3 μm for Y394F, and ± 0.5–3 μm for the Y186E, Y186F, and pCMF186 sets, respectively.

Motion correction and estimation of CTF parameters was done in cryoSPARC^54^ using patch motion and patch CTF correction routines. The results were curated manually and each data set was subject to standard image processing pipeline in cryoSPARC. Particles were extracted (to an iterative using a box with 320 to 420 px and twofold binning, resulting in a pixel size of 1.668 Å/px for Y394E sample and 1.566 Å/px for Y394F, Y186E, Y186F, and pCMF186 *h*Lon). Reference-free 2D classification was performed to remove unwanted particles and false positive picks. The selected particles were subjected to an ab initio routine using 3 classes for the initial 3D classification. The 3D-model representatives of the *h*Lon complex were chosen for subsequent Heterogeneous Refinement. Particles contributing to the best 3D models were selected, re-extracted and subjected to Non-uniform Refinement.

To create the initial model of the Y394E mutant, chain A was first extracted from an existing *h*Lon cryo-EM structure in the PDB (7NFY)^16^. This chain was cut into four separate domains—N-terminal (residues 123–414), ATPase (415–660), α-helical (661–750), and protease (751–948). The phased rotation function of MolRep (version 11.9.02)^55^ was used to fit six copies of each of the protease, α-helical, and ATPase domains into the cryo-EM potential map. The domains were fit sequentially in the given order. After six copies of each domain were fitted, phenix.real_space_refine^56^ was used to carry out rigid body refinement of each chain. The results of each domain search were input as a fixed model for subsequent domain searches. The N-terminal domain was too poorly resolved to be fit using individual domains, so the entire N-terminal domain was fit as a hexamer. After all domains had been fit, the individual domains were joined using Coot^57^ to make six chains. In this process, it was necessary to manually rebuild the loop containing residues 750–763, connecting the α-helical domain to the proteolytic domain. The fit of the residues to the map was then inspected using Coot and rebuilt where necessary. ADP was also modelled into all nucleotide binding pockets. The structure was refined in real space using PHENIX^58^.

For the remaining structures, the Y394E model was divided into an N-terminal domain unit and an ATPase-protease unit. The phased rotation function of MolRep was then used to fit one copy of each unit into the map of the given mutant. The units were then joined and rebuilding and refinement were carried out as before. This procedure was found to yield structures with slightly better geometry than the alternative. The final models were evaluated using MolProbity^59^. Details of the map reconstruction and model refinement statistics may be found in Supplementary Table S9.

Electrostatic surfaces were calculated using APBS^60^ and structural visualization was carried out using PyMOL^61^.

## Supplementary Information

Supplementary Information 1.

Supplementary Information 2.

Supplementary Information 3.

## Data Availability

All structures were deposited in the PDB with accession codes 8OJL (Y394E), 8OKA (Y394F), 8OM7 (Y186E), 8OVF (Y186F), and 8OVG (186pCMF). The cryo-EM maps are available either in the PDB or in the EMDB with accession codes EMD-16915 (Y394E), EMD-16923 (Y394F), EMD-16970 (Y186E), EMD-17213 (Y186F), and EMD-17214 (186pCMF).
