# Supplementary material for: Polyphosphate and tyrosine phosphorylation in the N-terminal domain of the human mitochondrial Lon protease disrupts its functions
Source: Sci Rep. 2024 Apr 30;14:9923. doi: 10.1038/s41598-024-60030-9 (PMC11061198; doi:10.1038/s41598-024-60030-9)
Supplement: Supplementary file 1 — Supplementary Information 1. [file 41598_2024_60030_MOESM1_ESM.pdf]

Polyphosphate and tyrosine phosphorylation in the N-terminal domain of the human mitochondrial Lon protease disrupts its functions.

Nina Kunová[1,2,+], Gabriela Ondrovičová[1,+], Jacob A. Bauer[1], Veronika Krajčovičová[1#], Matyáš Pinkas[3], Barbora Stojkovičová[1,2], Henrieta Havalová[1], Veronika Lukáčová[4], Lenka Kohútová[5], Július Košťan[6], Lucia Martináková[1], Peter Baráth[4,5], Jiří Nováček[3], Sebastian Zoll[7], Sami Kereïche[2,7]*, Eva Kutejová[1]*, and Vladimír Pevala[1]*

[1] Department of Biochemistry and Protein Structure, Institute of Molecular Biology, Slovak Academy of Sciences, Dúbravská cesta 21, 845 51 Bratislava, Slovakia

[2] Institute of Biology and Medical Genetics, First Faculty of Medicine, Charles University in Prague, Prague, Czech Republic

[3] CEITEC, Masaryk University in Brno, Brno, Czech Republic

[4] Medirex Group Academy, Nitra, Slovakia

[5] Institute of Chemistry, Slovak Academy of Sciences, Bratislava, Slovakia

[6] Department of Structural and Computational Biology, Max Perutz Labs, University of Vienna, Campus Vienna, Biocenter 5, Vienna, A-1030, Austria

[7] Institute of Organic Chemistry and Biochemistry of the Czech Academy of Sciences, Flemingovo namesti 542/2, 16000, Prague, Czech Republic

[#] Current address: Laboratory of Clinical and Molecular Genetics, National Institute of Children's Diseases, Limbová 1, 833 40, Bratislava, Slovakia

* vladimir.pevala@savba.sk; eva.kutejova@savba.sk; skere@lf1.cuni.cz

[+] these authors contributed equally to this work

SUPPLEMENTARY DATA

Table S1: Primers used for site-directed mutagenesis and PCR amplification.

Primer	Sequence 5′ → 3′
*h*Lon_Y186TAG_sense	GCCTGGATGAAATCTAGCACACGGGGACG
*h*Lon_Y186TAG_antisense	CGTCCCCGTGTGCTAGATTTCATCCAGGC
*h*Lon_Y394TAG_sense	GACCCACCGTAAGTAGCTGCTGCAGGAGC
*h*Lon_Y394TAG_antisense	GCTCCTGCAGCAGCTACTTACGGTGGGTC
*h*Lon_Y186E_sense	GAGCCTGGATGAAATCGAACACACGGGGACGTTTG
*h*Lon_Y186E_antisense	CAAACGTCCCCGTGTGTTCGATTTCATCCAGGCTC
*h*Lon_Y186F_sense	GCCTGGATGAAATCTTCCACACGGGGACGTTTG
*h*Lon_Y186F_antisense	CAAACGTCCCCGTGTGGAAGATTTCATCCAGGC
*h*Lon_Y394E_sense	CAGACCCACCGTAAGGAACTGCTGCAGGAGCAG
*h*Lon_Y394E_antisense	CTGCTCCTGCAGCAGTTCCTTACGGTGGGTCTG
*h*Lon_Y394F_sense	CAGACCCACCGTAAGTTCCTGCTGCAGGAGCAG
*h*Lon_Y394F_antisense	CTGCTCCTGCAGCAGGAACTTACGGTGGGTCTG

Table S2: Plasmid constructs used in this study.

Plasmid construct	Protein version	Host	Source
pProEx1-*h*Lon	*h*Lon (Δ1–114) lacking the import presequence with an N-terminal 6×His tag	*E. coli* Rosetta 2 (DE3)	gift of C. K. Suzuki
pEVOL	aminoacyl tRNA/aminoacyl-tRNA synthetase from *Methanocaldococcus jannaschii*	*E. coli* C321 (DE3)	gift of P. G. Schultz
pProEx1-*h*Lon_pCMF186	*h*Lon (Δ1–114) lacking the import presequence with a Y186X mutation and a 6×His tag	*E. coli* C321 (DE3)	this study
pProEx1-*h*Lon_pCMF394	*h*Lon (Δ1–114) lacking the import presequence with a Y394X mutation and a 6×His tag	*E. coli* C321 (DE3)	this study
pProEx1-*h*Lon_Y186E	*h*Lon (Δ1–114) lacking the import presequence with a Y186E mutation and a 6×His tag	*E. coli* Rosetta 2 (DE3)	this study
pProEx1-*h*Lon_Y186F	*h*Lon (Δ1–114) lacking the import presequence with a Y186F mutation and a 6×His tag	*E. coli* Rosetta 2 (DE3)	this study
pProEx1-*h*Lon_Y394E	*h*Lon (Δ1–114) lacking the import presequence with a Y394E mutation and a 6×His tag	*E. coli* Rosetta 2 (DE3)	this study
pProEx1-*h*Lon_Y394F	*h*Lon (Δ1–114) lacking the import presequence with a Y394F mutation and a 6×His tag	*E. coli* Rosetta 2 (DE3)	this study

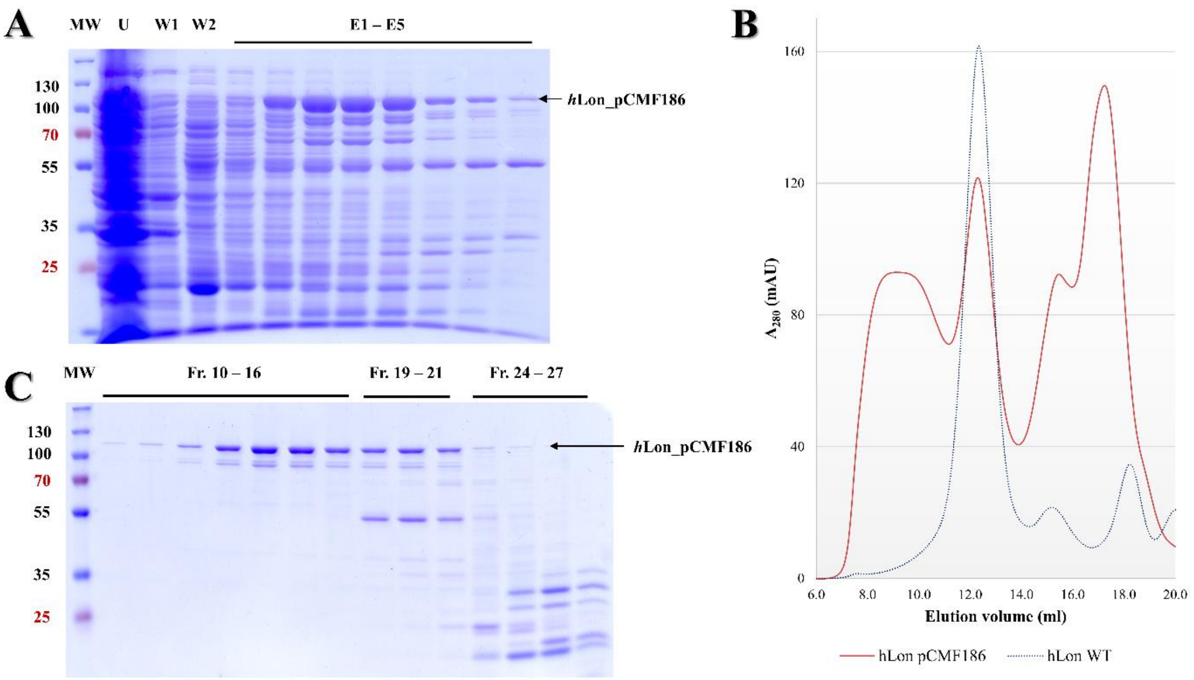

Figure S1. Isolation and purification of the phosphorylated human Lon protease variant *h*Lon_pCMF186. (**A**) Affinity chromatography of a 6×His-tagged recombinant *h*Lon_pCMF186 mutant on a NiNTA Agarose column (Qiagen). Elutions were made in a stepwise gradient of imidazole (0.1–0.5 M). (**B**) The sample was further purified by gel filtration on a Superose 6 10/300 GL column (GE Healthcare). A wild-type *h*Lon was used as a molecular mass standard. (**C**) The protein eluted in 11.5–13 ml (fractions 12–15) was pooled, and its concentration was determined using the BCA method. The samples from each purification step were separated in 12% SDS-polyacrylamide gels. MW – molecular weight marker; U – unbound fraction; W1, W2 – wash fractions; E – elutions; Fr. 10–27 – SEC fractions after the separation.

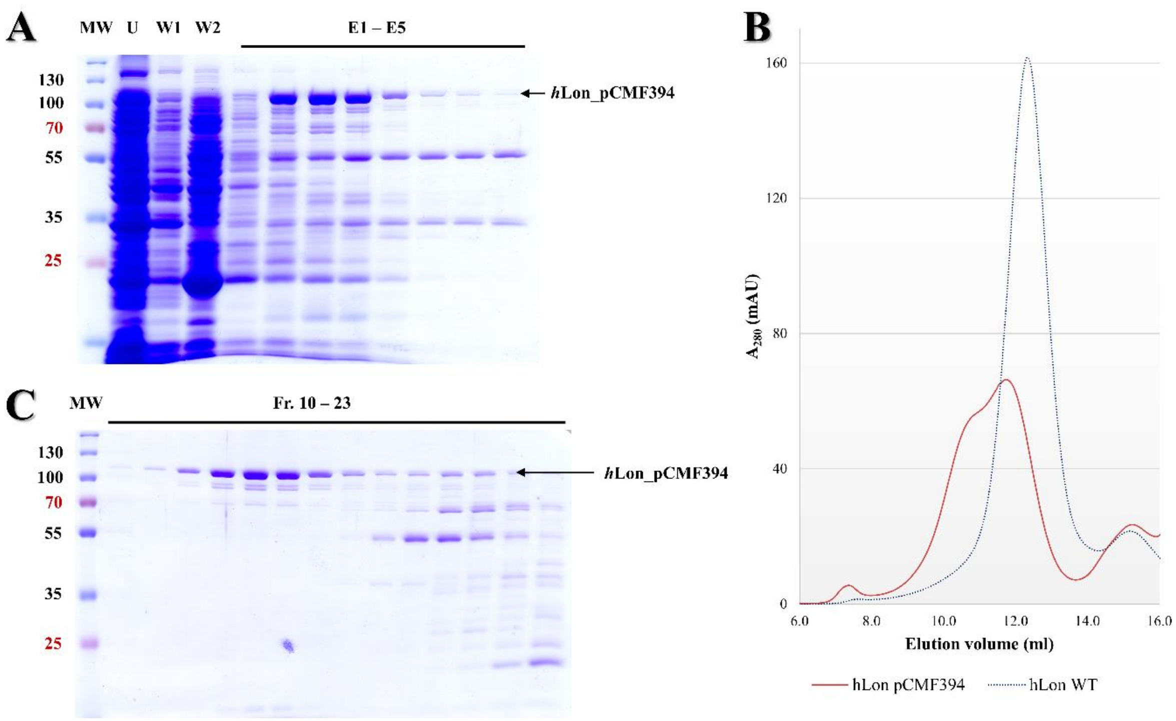

Figure S2. Isolation and purification of the phosphorylated human Lon protease variant *h*Lon_pCMF394. (**A**) Affinity chromatography of a 6×His-tagged recombinant *h*Lon_pCMF394 mutant on a NiNTA Agarose column (Qiagen). Elutions were made in a stepwise gradient of imidazole (0.1 – 0.5 M). (**B**) The sample was further purified by gel filtration on a Superose 6 10/300 GL column (GE Healthcare). Wild-type *h*Lon was used as a molecular mass standard. (**C**) The protein eluted in 11.5–13 ml (fractions 12–15) was pooled, and its concentration was determined using the BCA method. The samples from each purification step were separated in 12% SDS-polyacrylamide gels. MW – molecular weight marker; U – unbound fraction; W1, W2 – wash fractions; E – elutions; Fr. 10–23 – SEC fractions after the separation.

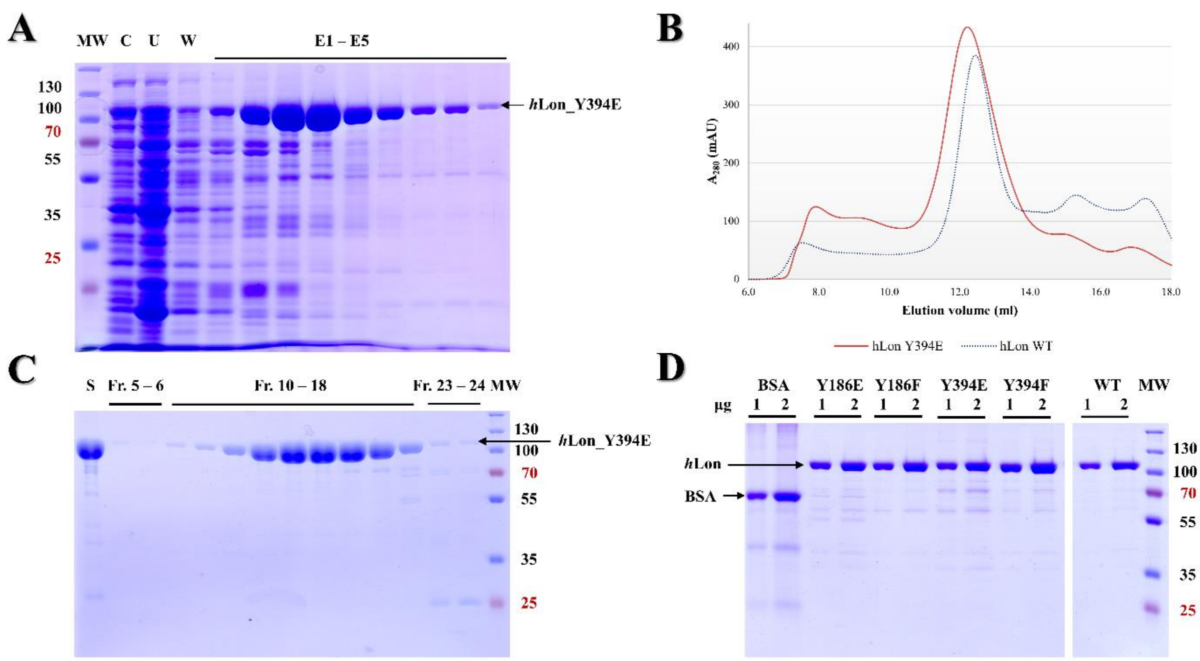

Figure S3. Isolation and purification of the phosphorylation-mimicking human Lon protease versions *h*Lon_Y186E and *h*Lon_Y394E, and the control versions *h*Lon_Y186F and *h*Lon_Y394F. (**A**) Affinity chromatography of a 6×His-tagged recombinant *h*Lon_Y394E mutant on a NiNTA Agarose column (Qiagen). Elutions were made in a stepwise imidazole gradient (0.1–0.5 M). (**B**) The sample was further purified by gel filtration on a Superose 6 10/300 GL column (GE Healthcare). Wild-type *h*Lon was used as a molecular mass standard. (**C**) The protein eluted in 11–13 ml (fractions 11–15) was pooled, and its concentration was determined using the BCA method. (**D**) Finally, the concentration of the phospho-mimicking *h*Lon mutants and wild type *h*Lon was compared to a known amount of BSA (1 µg – lane 1; 2 µg – lane 2). The samples from each purification step were separated in 12% SDS-polyacrylamide gels. MW – molecular weight marker; C - cytosolic fraction; U – unbound fraction; W – wash fraction; E – elutions; S – sample loaded onto a column; Fr. 5–24 – SEC fractions after the separation; BSA – bovine serum albumin; Y186E, Y394E – phosphorylation-mimicking *h*Lon mutants; Y186F, Y394F – control *h*Lon mutants; WT – wild type *h*Lon.

Table S3: (in a separate *.xls file). **The list of proteins and pCMF sites.** The table lists protein groups and pCMF-modified sites identified in *h*Lon_pCMF186 and *h*Lon_ pCMF394 samples detected by the MS-MS analyses.

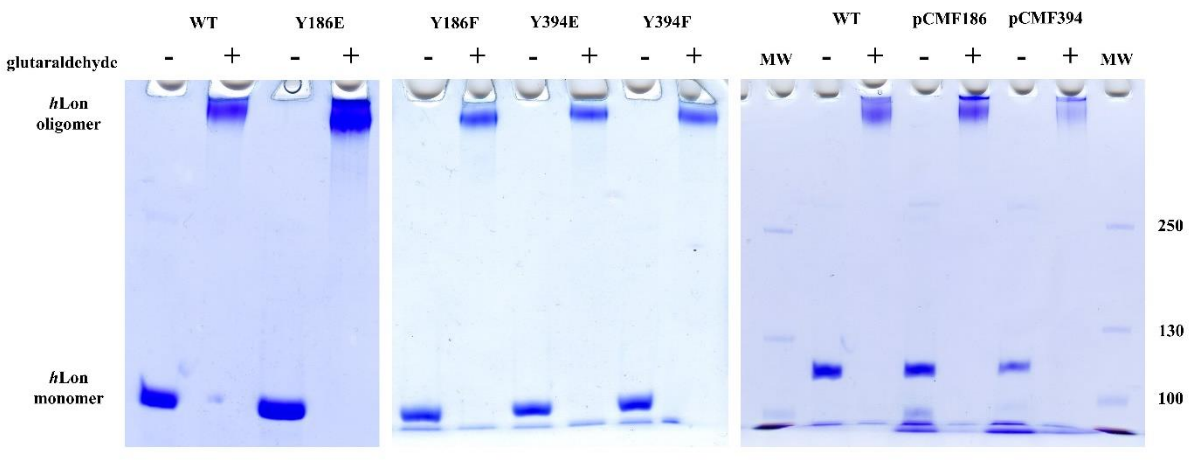

Figure S4. Crosslinking of wild-type *h*Lon and its variants prepared in this study. A given amount of protein was crosslinked with 0.1% (v/v) glutaraldehyde for 60 min at room temperature and then separated on a 5% SDS-PAGE gel. All *h*Lon proteins form oligomeric structures in the presence of a cross-linker (indicated with +). MW – molecular weight marker; pCMF186, pCMF394 – phosphorylated *h*Lon variants; Y186E, Y394E – phosphorylation-mimicking *h*Lon mutants; Y186F, Y394F – control *h*Lon mutants; WT – wild-type *h*Lon.

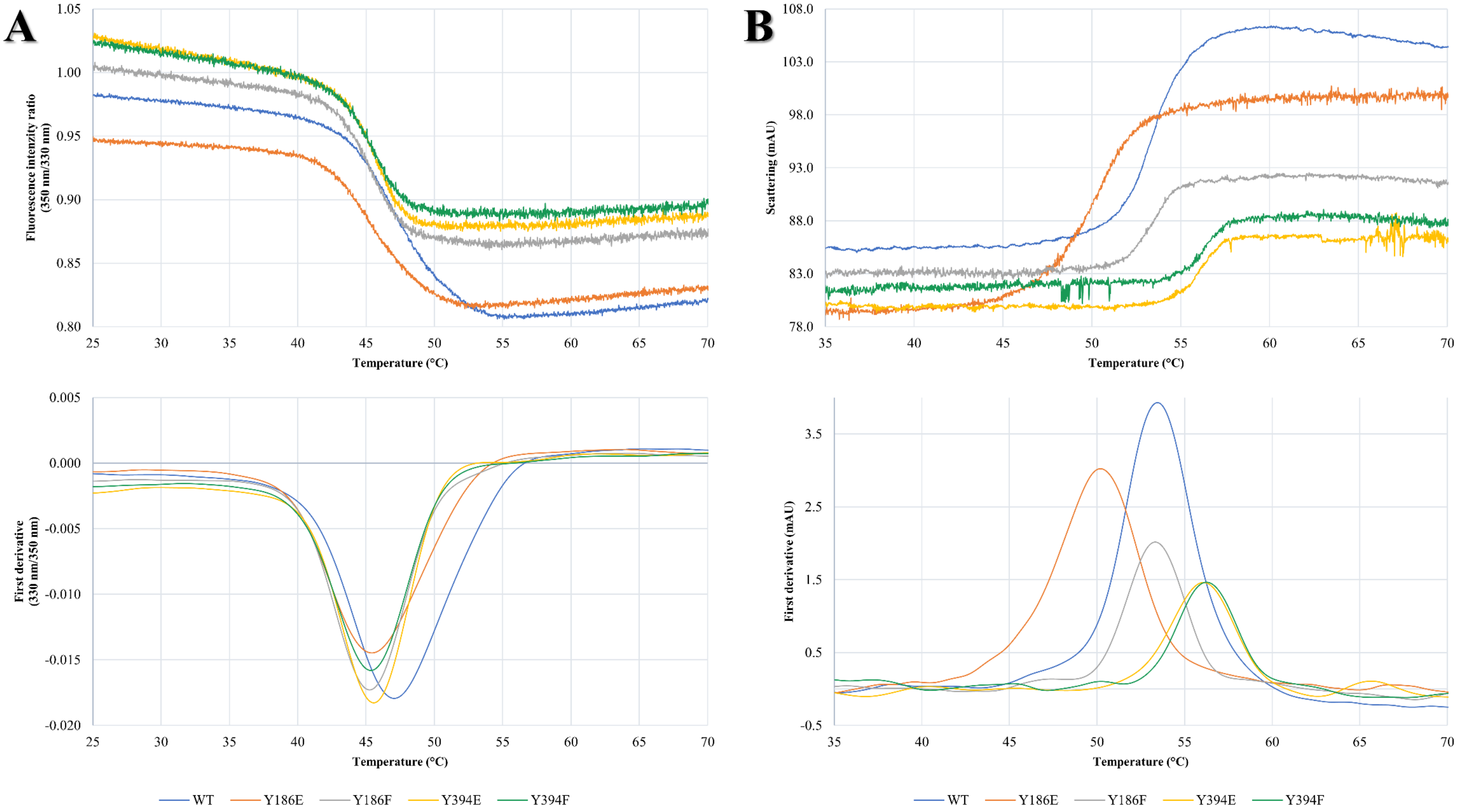

Figure S5. NanoDSF thermograms showing the effect of *h*Lon Tyr186 and *h*Lon Tyr394 replacement with either Glu (Y186E, Y394E) or Phe (Y186F, Y394F) on the propensity for *h*Lon to unfold and aggregate. (**A**) Temperature dependence of a fluorescence intensity ratio (350 nm/330 nm) and the first derivative of the fluorescence intensity ratio (*d*(350 nm/330 nm)/*d*T) showing the unfolding of *h*Lon variants at a concentration of 0.3 µg/µl in 40 mM HEPES, pH 8.0; 150 mM NaCl; 5% (v/v) glycerol. (**B**) Light scattering thermograms showing the aggregation of *h*Lon variants.

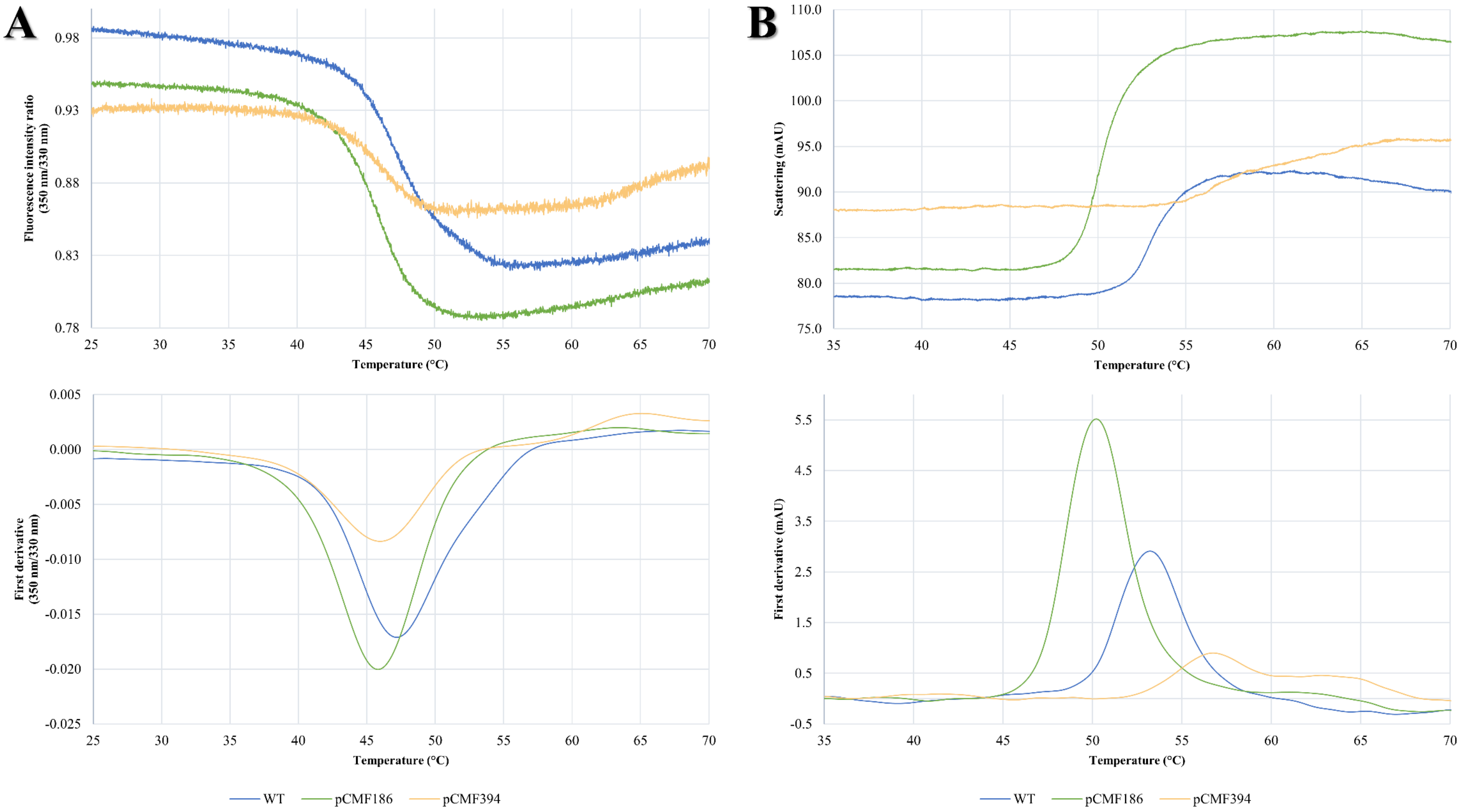

Figure S6. NanoDSF thermograms showing the effect of *h*Lon Tyr186 and *h*Lon Tyr394 replacement with *para*-carboxymethyl-L-phenylalanine (pCMF) on the propensity for *h*Lon to unfold and aggregate. (**A**) Temperature dependence of a fluorescence intensity ratio (350 nm/330 nm) and the first derivative of the fluorescence intensity ratio (*d*(350 nm/330 nm)/*d*T) showing the unfolding of *h*Lon mutants at a concentration of 0.3 µg/µl in 40 mM HEPES, pH 8.0; 150 mM NaCl; 5% (v/v) glycerol. (**B**) Light scattering thermograms showing the aggregation of *h*Lon variants.

Table S4: The denaturation temperatures (T_m) and aggregation onset temperatures (T_{onset}) of non-phosphorylated and phosphorylation-mimicking *h*Lon variants compared to the wild-type *h*Lon. These were determined by NanoDSF and the individual measurements are shown in Supplementary Figures S5 and S6.

Sample	Inflection point for ratio (T_m)	Onset of aggregation (T_{onset})
WT	50.5 ± 0.3 °C	56.6 ± 0.8 °C
Y186E	47.8 ± 0.1 °C	50.2 ± 0.5 °C
Y186F	50.3 ± 0.3 °C	56.7 ± 0.5 °C
pCMF186	45.2 ± 0.9 °C	48.6 ± 2.8 °C
Y394E	48.4 ± 0.1 °C	59.0 ± 0.4 °C
Y394F	49.0 ± 0.1 °C	56.9 ± 1.0 °C
pCMF394	45.4 ± 0.6 °C	53.1 ± 0.9 °C

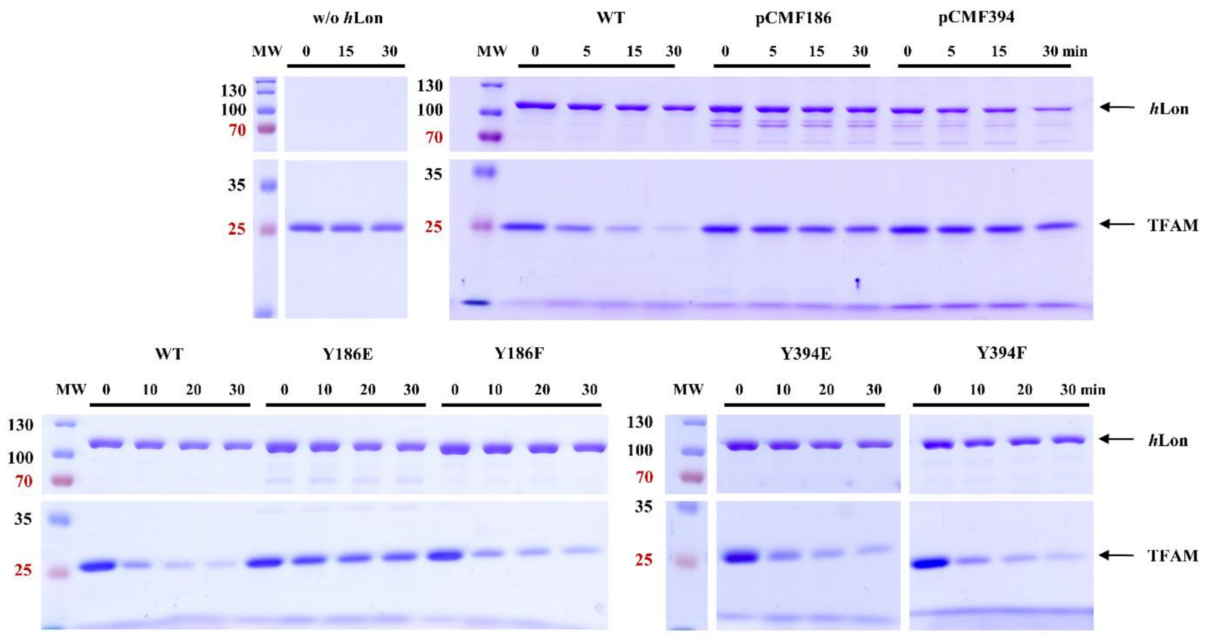

Figure S7. TFAM digested by the phosphorylated and phosphorylation-mimicking *h*Lon variants. (A) 1 μg TFAM was incubated with 1 μg of the given *h*Lon variant in reactions containing 2 mM ATP and 10 mM $MgCl_2$. Samples were withdrawn at the indicated times and loaded on a 12% SDS-polyacrylamide gel.

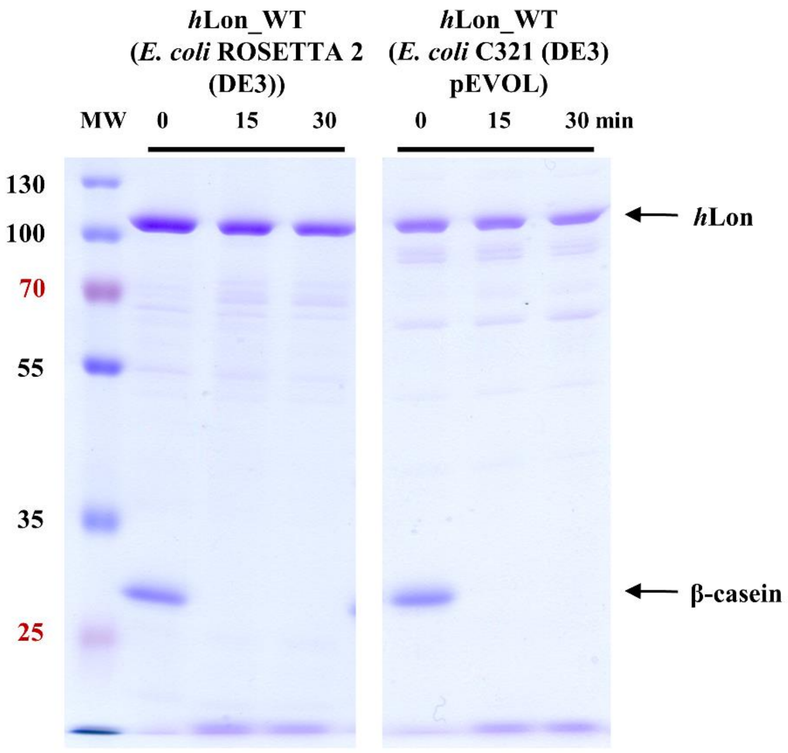

Figure S8. Comparison of β-casein digestion by wild-type *h*Lon expressed in different bacterial backgrounds. Again, 1 µg β-casein was incubated with 1 µg wild-type *h*Lon in reactions containing 2 mM ATP and 10 mM $MgCl_2$. Samples were withdrawn at the indicated times and loaded on a 12% SDS-polyacrylamide gel. β-casein was digested by wild-type *h*Lons produced in either *E. coli* Rosetta 2 (DE3) or *E. coli* C321 (DE3) both transformed with a pEVOL plasmid designed for the incorporation of pCMF used to produce the phosphorylated *h*Lon variants (see Methods). MW – molecular weight marker; WT – wild-type *h*Lon.

Table S5: (in a separate *.xls file). **The list of TFAM peptides.** The table lists all identified TFAM peptides produced in digestion experiments with *h*Lon_pCMF394 and *h*Lon_WT detected by LC-MS/MS analyses. TFAM peptides with score greated than 100 were used for visualization of a cleavage specificity of *h*Lon versions used in Figure 3.

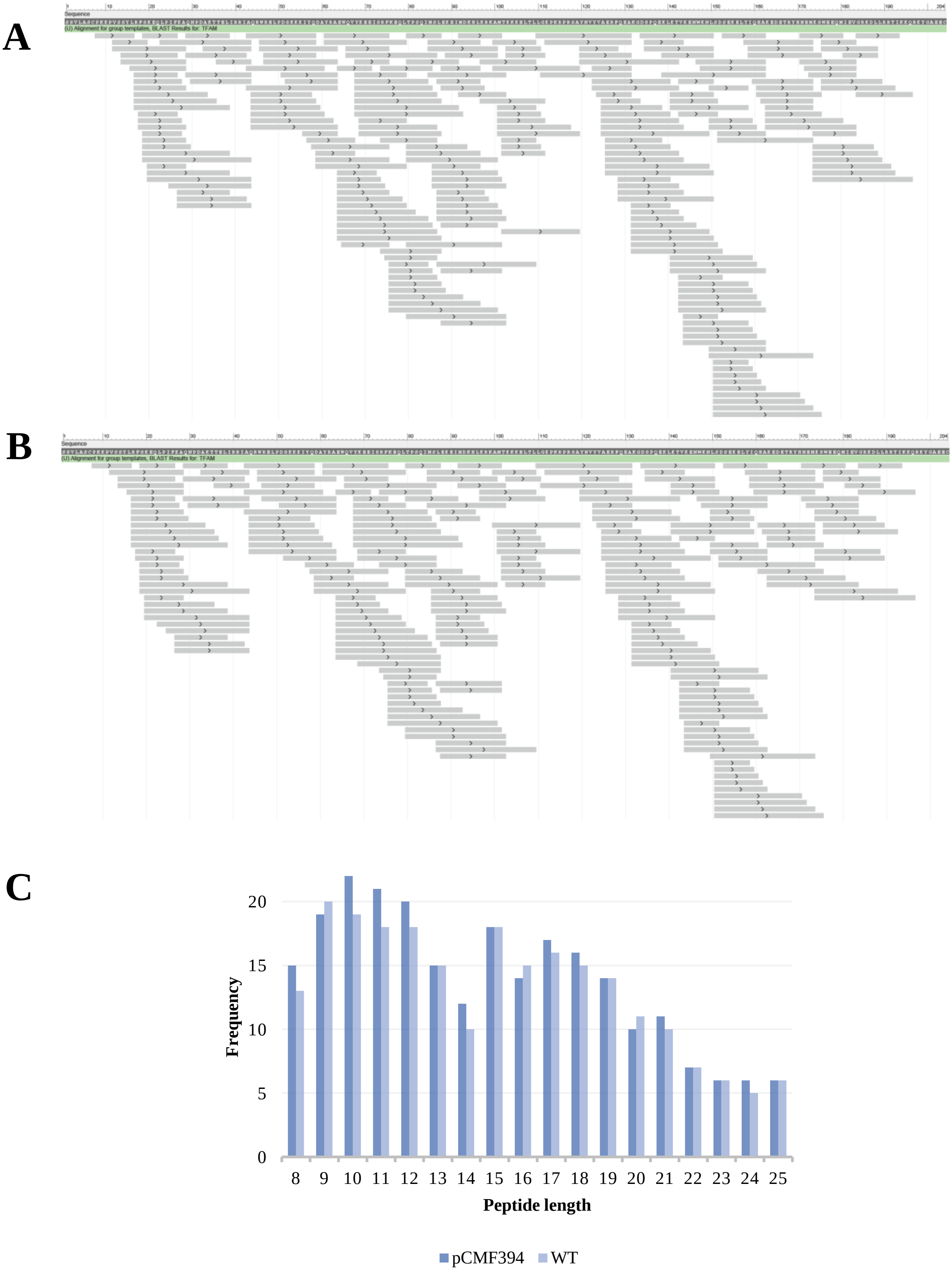

Figure S9. Peptides generated by the degradation of TFAM by *h*Lon pCMF394 (A) and *h*Lon WT (B). A graphic representation of TFAM peptides (horizontal gray lines) produced by *h*Lon-mediated digestion as aligned to the mature TFAM sequence (residues 43–246; marked as 1–204 here). Only peptides with scores greater than 100 identified by MS/MS in at least one biological replicate were considered. (**C**) A histogram comparing the peptide length distribution frequency between the pCMF394 and the wild-type *h*Lon variants.

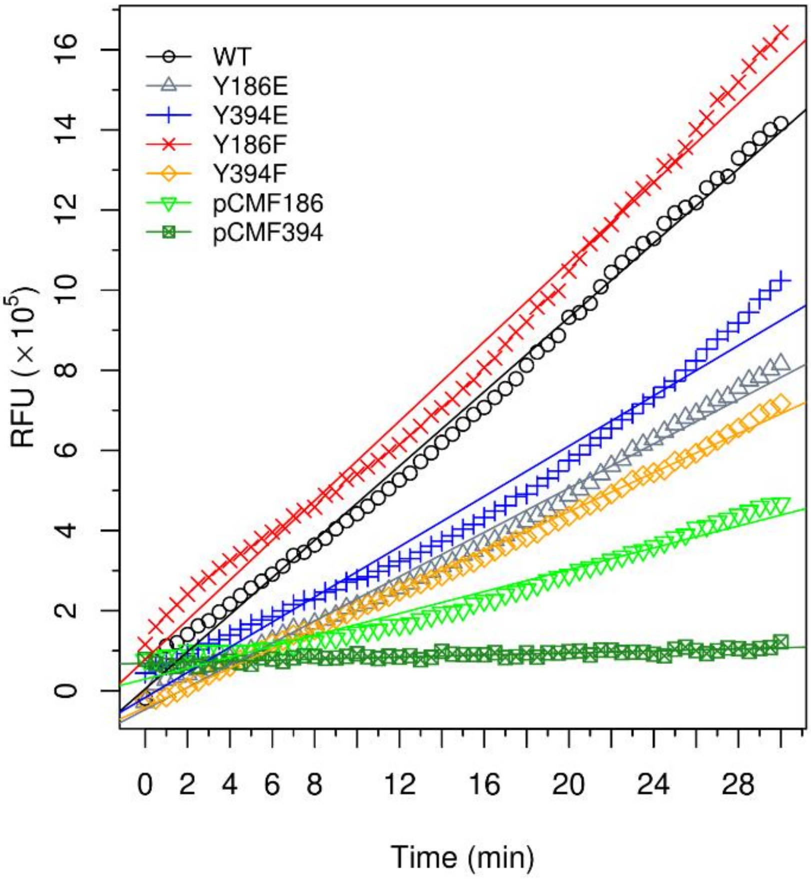

Figure S10. Protease activity of phosphorylated and phosphorylation-mimicking *h*Lon variants against FITC-casein. Purified *h*Lon variants (5 μg) were incubated with 7.5 μg FITC-casein at 37°C in a reaction mixture containing 0.5 mM ATP and 40 mM $MgCl_2$. The fluorescence triggered by FITC-casein digestion was measured (excitation wavelength 487 nm, emission wavelength 528 nm) every 30 seconds over a 30-minute span. The values show the average of at least three independent measurements. WT – wild-type *h*Lon; pCMF186, pCMF394 – phosphorylated *h*Lon variants; Y186E, Y394E – phosphorylation-mimicking *h*Lon mutants; Y186F, Y394F – control *h*Lon mutants; RFU – relative fluorescence unit.

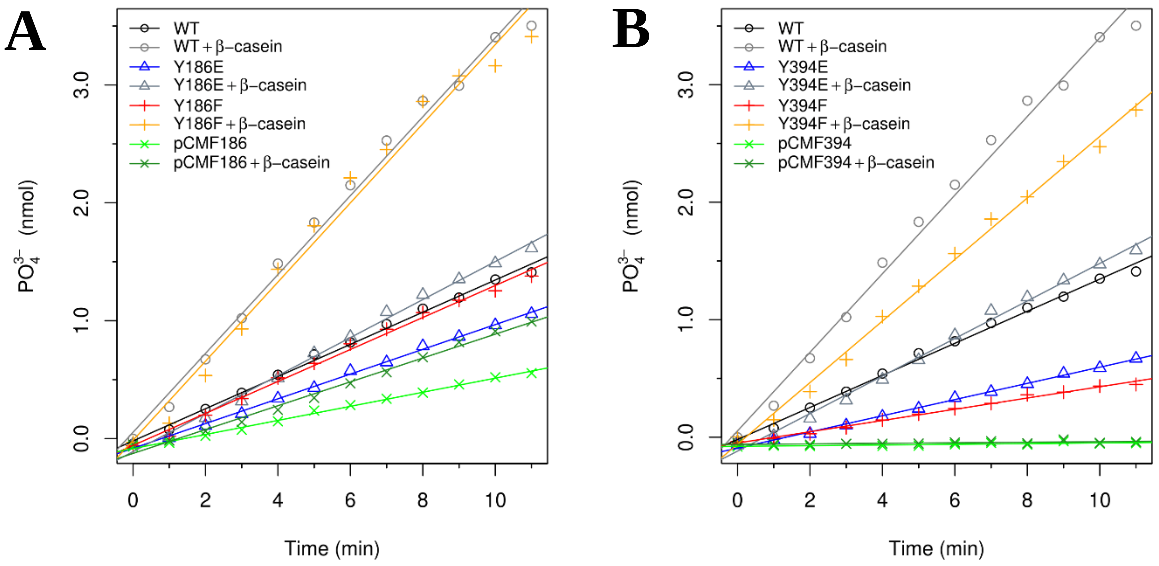

Figure S11. ATPase activity of phosphorylated and phosphorylation-mimicking *h*Lon variants mutated in Tyr186 (A) or Tyr394 (B). Purified *h*Lon variants (5 µg) were incubated either alone or in the presence of 25 µg β-casein at 37°C in a reaction mixture containing 0.5 mM ATP, 40 mM $MgCl_2$ and a colorimetric agent. The absorbance of the colored complex formed was measured at 660 nm every 60 seconds over a 12-minute span. The plots show the average of at least three independent measurements. WT – wild-type *h*Lon; pCMF186, pCMF394 – phosphorylated *h*Lon variants; Y186E, Y394E – phosphorylation-mimicking *h*Lon mutants; Y186F, Y394F – control *h*Lon mutants; PO_4^3 – phosphate.

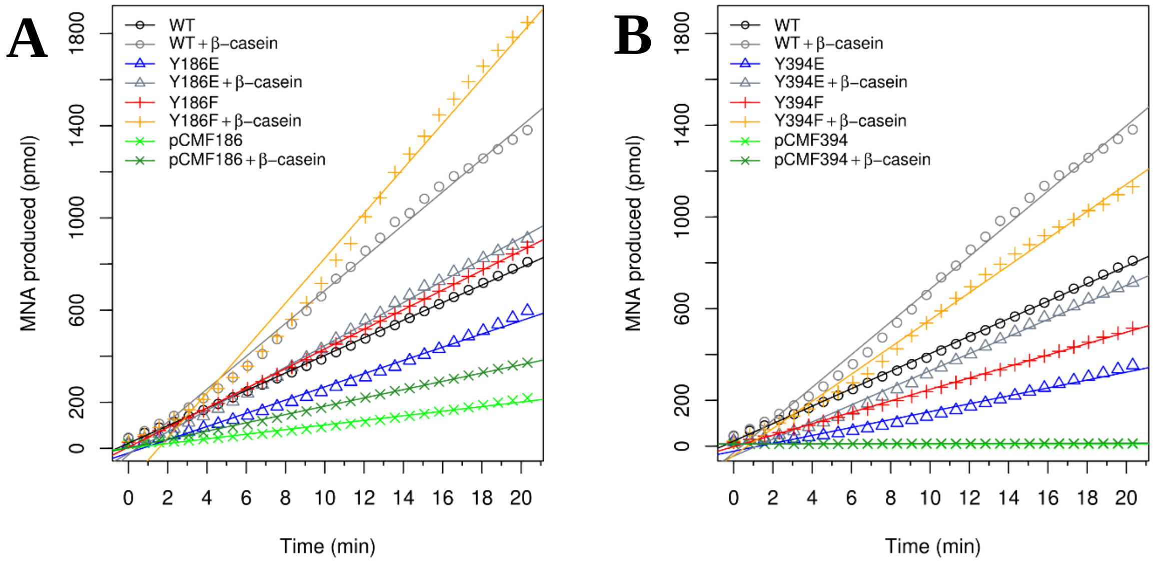

Figure S12. Peptidase activity of phosphorylated and phosphorylation-mimicking *h*Lon variants mutated in Tyr186 (A) or Tyr394 (B) with GAAF-MNA. Purified *h*Lon variants (5 µg) were incubated either alone or in the presence of 25 µg β-casein at 37°C in a reaction mixture containing 0.5 mM ATP, 40 mM $MgCl_2$ and 0.25 mM GAAF-MNA. The fluorescence triggered by GAAF-MNA cleavage was measured (excitation wavelength 340 nm, emission wavelength 440 nm) every 45 seconds over a 20-minute span. The values show the average of at least three independent measurements. WT – wild-type *h*Lon; pCMF186, pCMF394 – phosphorylated *h*Lon variants; Y186E, Y394E – phosphorylation-mimicking *h*Lon mutants; Y186F, Y394F – control *h*Lon mutants; MNA – methoxy-β-naphthylamide; GAAF-MNA – glutaryl-Ala-Ala-Phe-4-methoxy-β-naphthylamide.

Table S6: ATPase activities comparing wild-type *h*Lon and quintupled amount of the pCMF394 variant. The values of Frac. Basal and Frac. Stimulated were not normalized to the amount of *h*Lon WT. Values are ±1 standard error. nM.min^{-1} – nanomoles of PO_4^{3-} released by *h*Lon per minute. Frac. Basal – ratio of the basal activity of the mutant to basal activity of the WT; Frac. Stimulated – ratio of the stimulated activity of the mutant to stimulated activity of the WT.

		ATPase activity (nmol·min^{-1})				
**Protein**	**Molar conc. (nM)**	**Basal**	**Stimulated**	**Stimulation**	**Frac. Basal**	**Frac. Stimulated**
**WT**	28	0.137 ± 0.003	0.330 ± 0.010	2.409	1.000	1.000
**pCMF394**	140	0.025	0.032	1.280	0.18	0.097

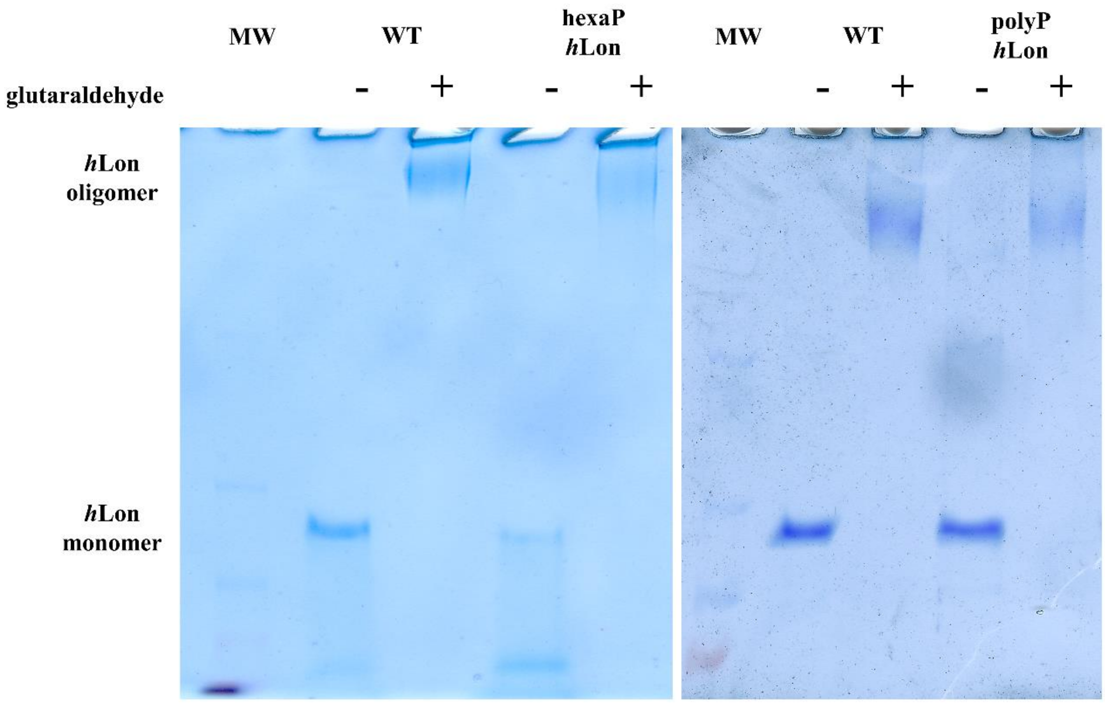

Figure S13. Crosslinking of hexaphosphate- and polyphosphate-treated *h*Lon. A given amount of *h*Lon was crosslinked with 0.1% (v/v) glutaraldehyde for 60 min at room temperature and separated on a 5% SDS-PAGE gel. The samples incubated in the presence of a cross-linker are indicated by +. The positions of *h*Lon oligomeric and monomeric states are shown. MW – molecular weight marker; WT – wild-type *h*Lon; hexaP *h*Lon – hexaphosphate-treated *h*Lon; polyP *h*Lon – polyphosphate-treated *h*Lon.

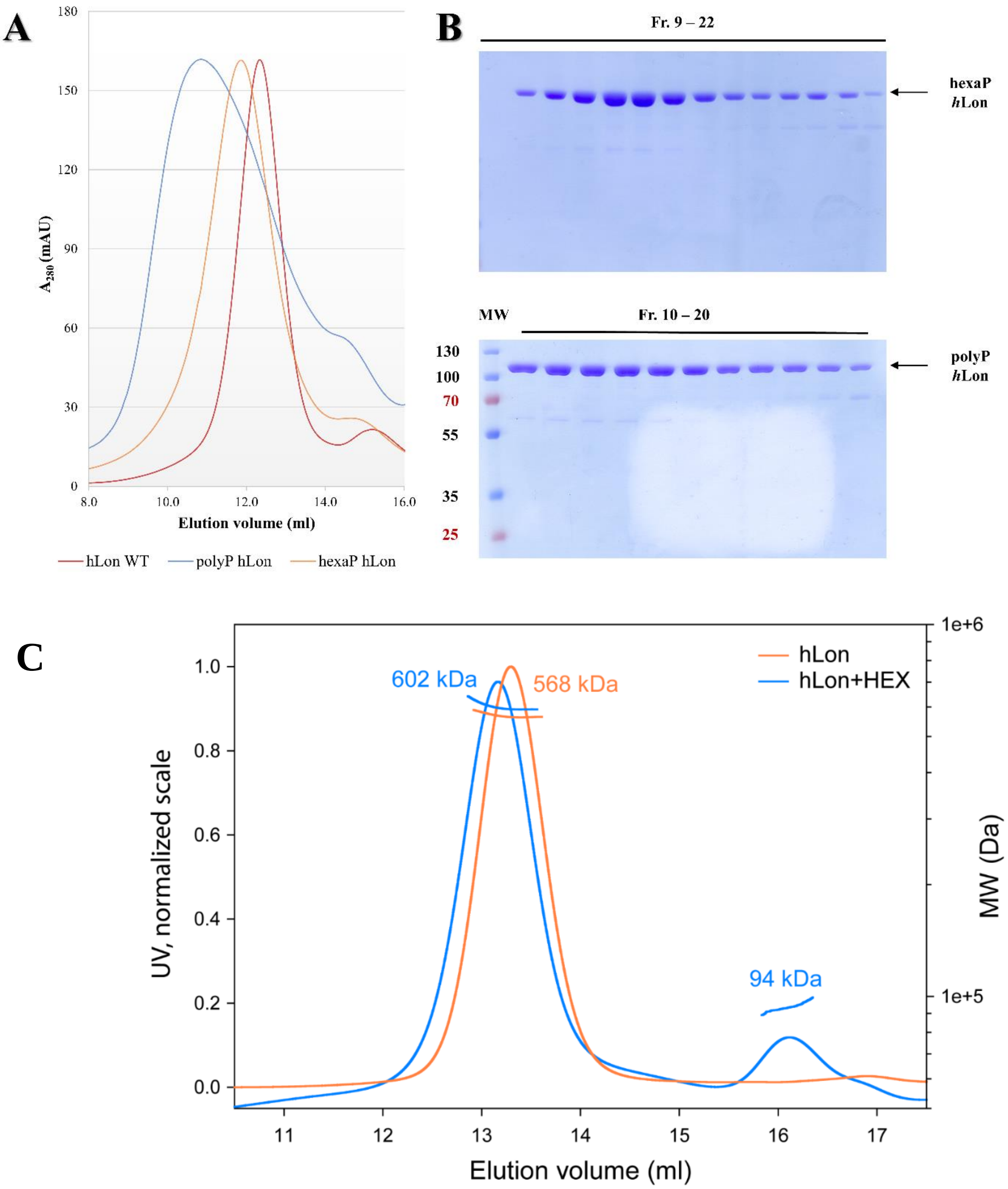

Figure S14. Preparation of hexaphosphate-treated (hexaP) and polyphosphate-treated (polyP) *h*Lon. (**A**) Purified wild-type *h*Lon (colored red) was loaded on a Superose 6 10/300 GL column (GE Healthcare). The peak fractions (11.5–13 ml; fractions 12–15) were pooled, concentrated and incubated either with 10 mM hexaphosphate or 10 mM polyphosphate at 37°C for 2 hours. Afterwards, the polyP- and hexaP-treated samples (colored yellow and blue) were separated on the same column. (**B**) The fractions eluted in 11.0–12.5 ml (Fr. 10–13) of a hexaP-treated sample and the fractions eluted in 10.0–11.5 ml (Fr. 12–15) of a polyP-treated sample were pooled, concentrated and subjected to further analyses. The samples from each purification step were separated in 12% SDS-polyacrylamide gels.

MW – molecular weight marker; Fr. 9–22 and Fr. 10–20 – SEC fractions after the separation. (**C**) Molecular weight determination of a hexaP-treated *h*Lon by SEC-MALLS. The samples were separated on a Superose 6 Increase 10/300 GL column (Cytiva) in 40 mM HEPES, pH 8.0, 150 mM NaCl, 5% (v/v) glycerol. The given SEC elution profile has been normalized by sample concentration. The line crossing the chromatogram profile shows the molecular mass of the protein determined by SEC-MALLS and refractive index detection apparatus. The analysis of light scattering intensity at different angles and refractive indices as a function of elution volume show that the molecular weight of a hexaP-modified hexameric *h*Lon is 602 kDa and that of a hexaP-modified monomeric *h*Lon version is 94 kDa (colored blue). The theoretical molecular weight corresponding to a wild-type *h*Lon hexamer is 568 kDa (colored orange).

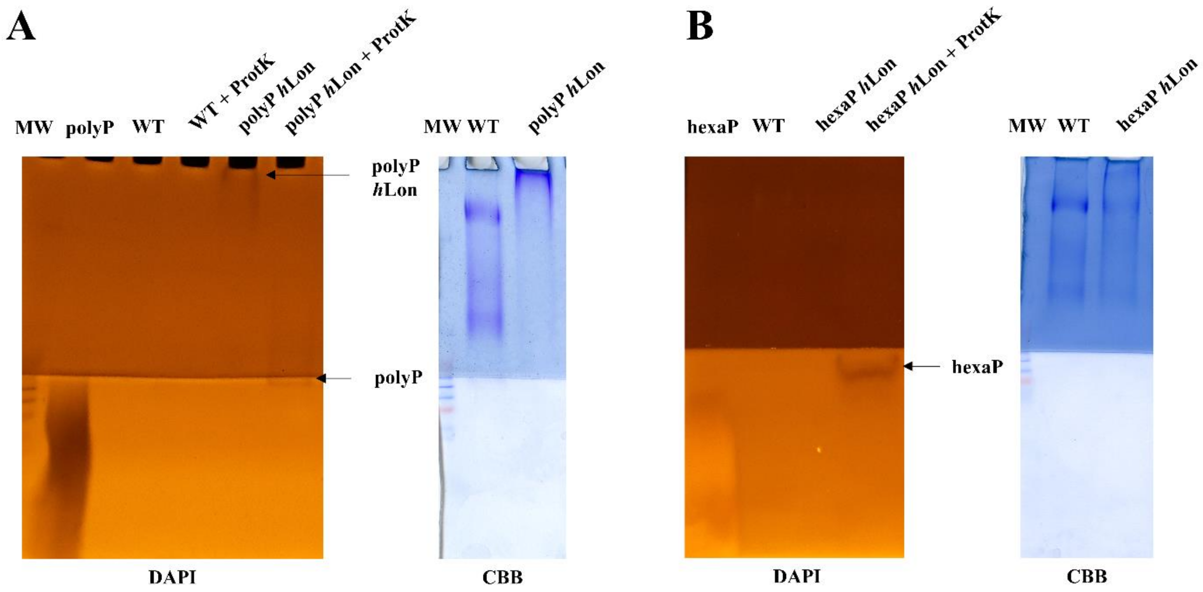

Figure S15. Native PAGE of polyphosphate- and hexaphosphate-treated *h*Lons. A given amount of polyphosphate-treated (**A**) and hexaphosphate-treated (**B**) *h*Lon samples were incubated with Proteinase K for 10 minutes at 30°C and loaded onto native 6–20 % polyacrylamide gels stained in DAPI (20 µg/ml) and Coomassie Brilliant Blue (CBB). MW – molecular weight marker; WT – wild-type *h*Lon; hexaP – hexaphosphate; polyP – polyphosphate; ProtK – Proteinase K.

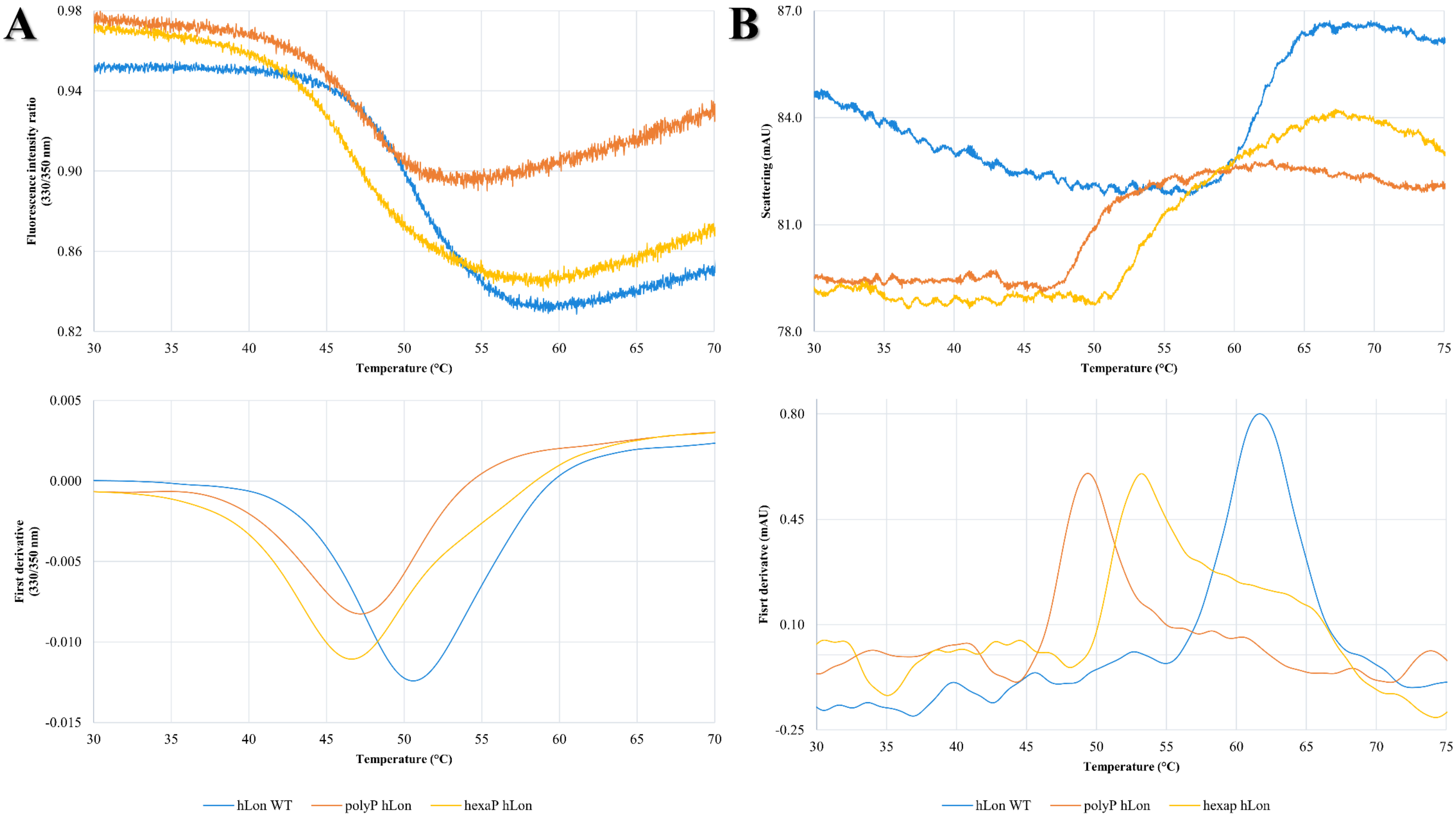

Figure S16. NanoDSF thermograms showing the effect of *h*Lon hexa- and poly-phosphorylation on the propensity for *h*Lon to unfold and aggregate. (**A**) Temperature dependence of a fluorescence intensity ratio (350 nm/330 nm) and the first derivative of the fluorescence intensity ratio (*d*(350 nm/330 nm)/*d*T) showing the unfolding of phosphate-treated *h*Lon at a concentration of 0.3 μg/μl in 40 mM HEPES, pH 8.0; 150 mM NaCl; 5% (v/v) glycerol. (**B**) Light scattering thermograms showing the aggregation of hexa- and poly-phosphorylated *h*Lon.

Table S7: The denaturation temperatures (T_m) and aggregation onset temperatures (T_{onset}) of polyphosphate-treated *h*Lon variants compared to the wild-type *h*Lon. These were determined by NanoDSF and the individual measurements are shown in Supplementary Figure S14.

Sample	Inflection point for ratio (T_m)	Onset of aggregation (T_{onset})
WT	50.5 ± 0.3 °C	56.6 ± 0.8 °C
hexaP *h*Lon	47.7 ± 0.2 °C	51.6 ± 0.2 °C
polyP *h*Lon	46.9 ± 0.7 °C	44.7 ± 0.6 °C

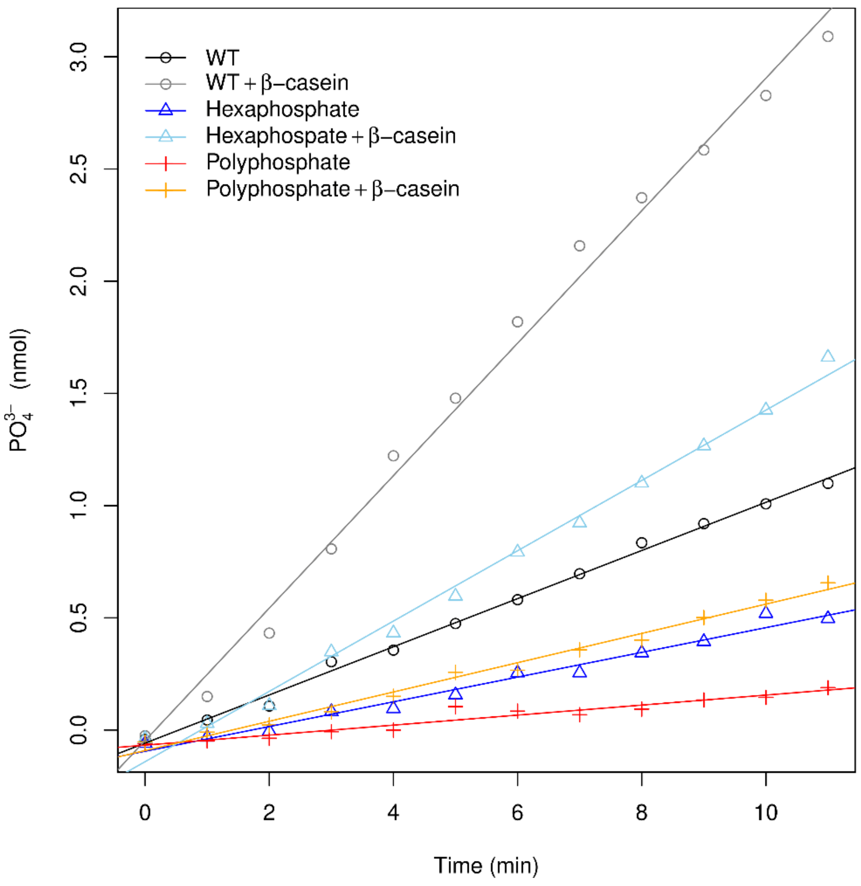

Figure S17. Enzymatic properties of hexaphosphate-treated and polyphosphate-treated *h*Lon variants compared to the wild-type *h*Lon protease. Basal and β-casein-stimulated ATPase activities of hexaP *h*Lon, polyP *h*Lon and WT were measured as the amount of phosphate (PO_4^{3-}) released in *h*Lon-mediated ATP hydrolysis as described in Ambro *et al.*[1]. The values show the average of at least three independent measurements. WT – wild-type *h*Lon.

Table S8: ATPase activities of hexaphosphate- and polyphosphate-treated *h*Lon compared to the wild-type. Values are ±1 standard error. nM·min^{-1} – nanomoles of PO_4^{3-} released by *h*Lon per minute. Frac. Basal – ratio of the basal activity of the mutant to basal activity of the WT; Frac. Stimulated – ratio of the stimulated activity of the mutant to stimulated activity of the WT; WT – wild-type *h*Lon; hexaP – hexaphosphate-treated *h*Lon; polyP – polyphosphate-treated *h*Lon.

	ATPase activity (nmol·min^{-1})				
**Protein**	**Basal**	**Stimulated**	**Stimulation**	**Frac. Basal**	**Frac. Stimulated**
**WT**	0.107 ± 0.002	0.295 ± 0.008	2.757	1.000	1.000
**hexaP**	0.055 ± 0.003	0.157 ± 0.004	2.854	0.514	0.532
**polyP**	0.022 ± 0.002	0.065 ± 0.002	2.954	0.206	0.220

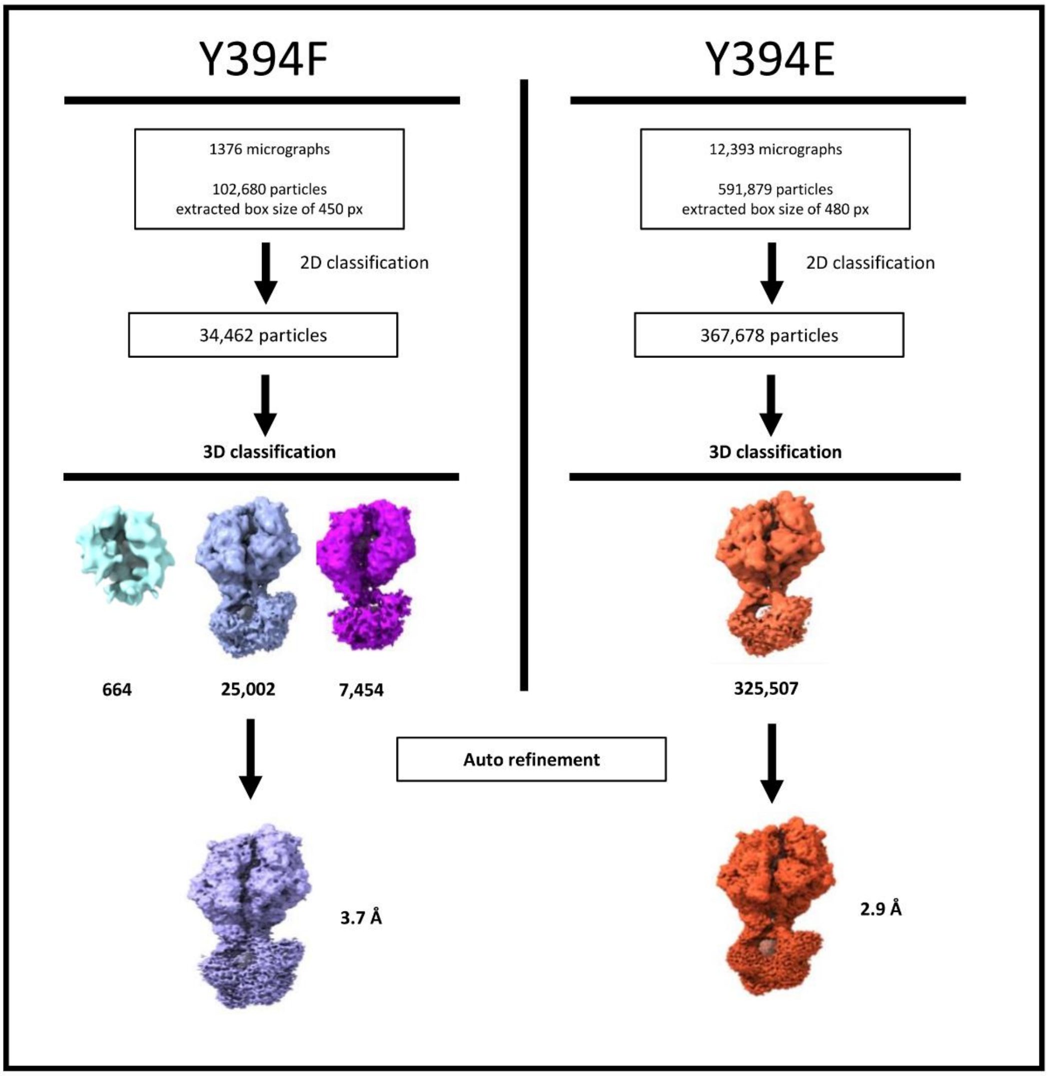

Figure S18. Flowchart of Cryo-EM data processing. Both datasets were processed in parallel using Cryosparc V4.0.1[2].

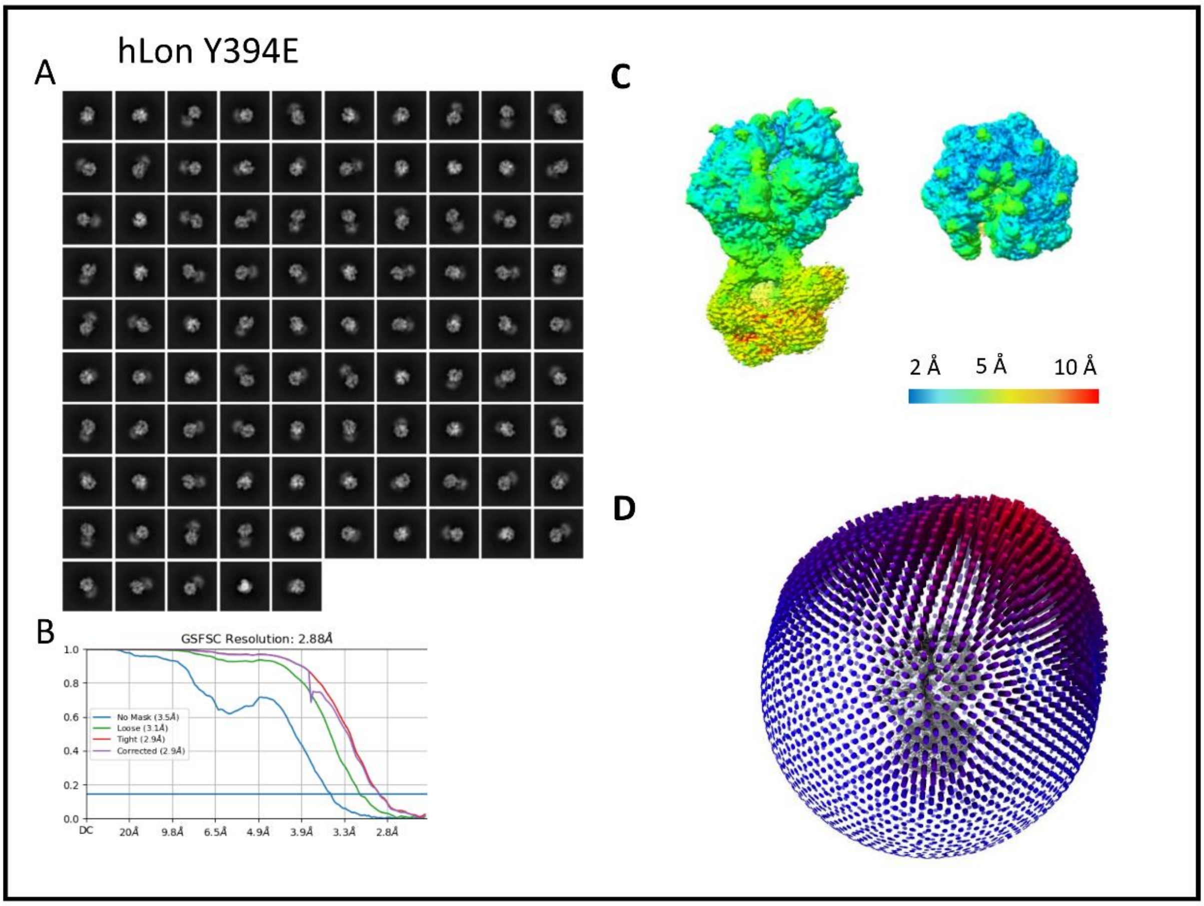

Figure S19. Cryo-EM data collection and 3D map reconstruction for *h*Lon_Y394E. (**A**) Overview of the selected 2D classes. (**B**) Gold-standard (0.143) Fourier shell correlation curves (FSC) for the EM maps. (**C**) Local resolution maps. Although the overall resolution according to the FSC plot is 2.88 Å, the NTD reaches only 8–10 Å. (**D**) Angular distribution of particles for final reconstruction. Each cylinder is proportional to the number of particles for that view.

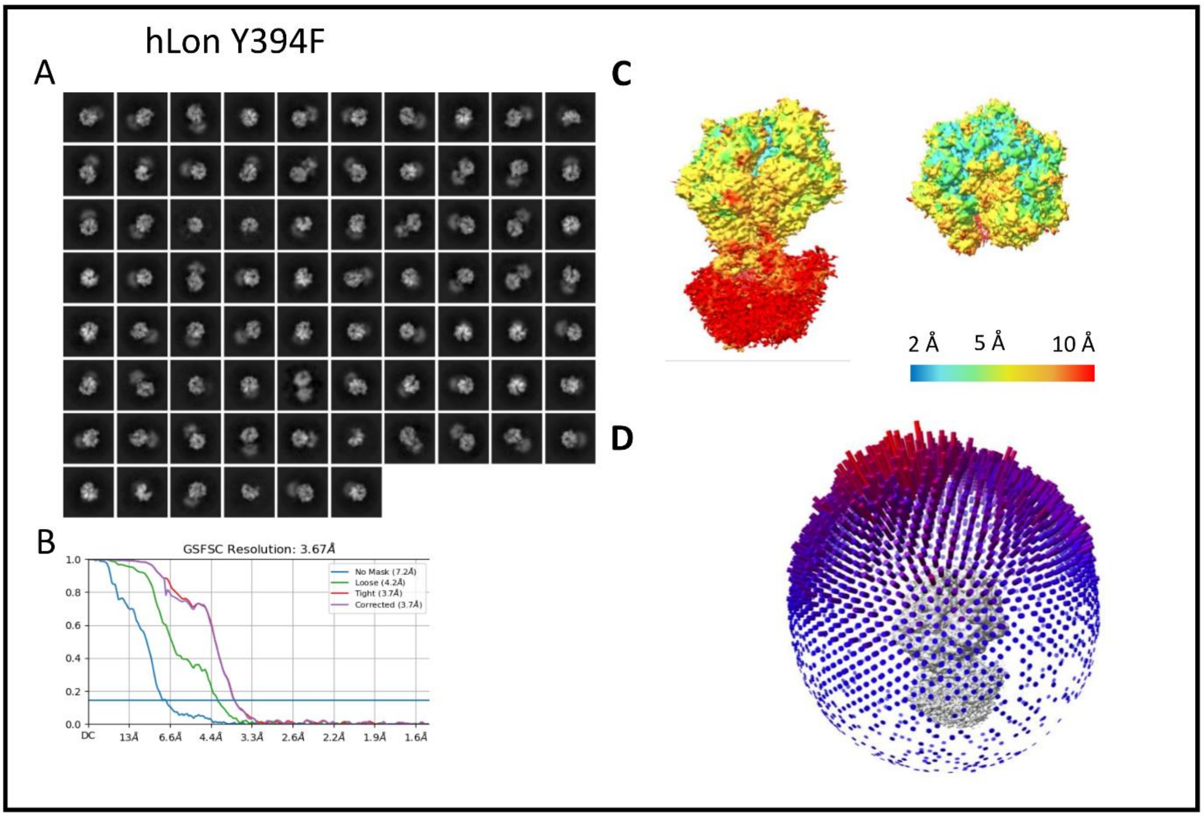

Figure S20. Cryo-EM data collection and 3D map reconstruction for *h*Lon_Y394F. (**A**) Overview of the selected 2D classes. (**B**) Gold-standard (0.143) Fourier shell correlation curves (FSC) for the EM maps. (**C**) Local resolution maps. Although the overall resolution according to the FSC plot is 3.67 Å, the NTD reaches only 10 Å. (**D**) Angular distribution of particles for final reconstruction. Each cylinder is proportional to the number of particles for that view.

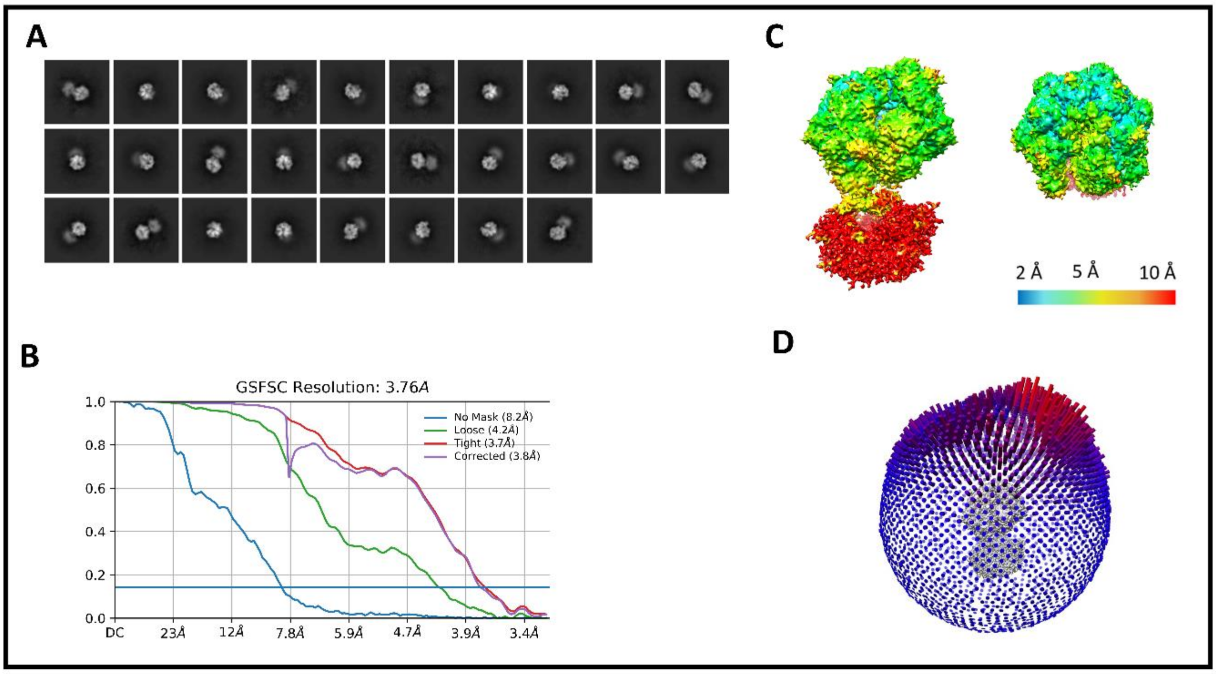

Figure S21. CryoEM data collection and 3D map reconstruction for *h*Lon_Y186E. (**A**) Overview of the selected 2D classes. (**B**) Gold-standard (0.143) Fourier shell correlation curves (FSC) for the EM maps. (**C**) Local resolution maps. Although the overall resolution according to the FSC plot is 3.76 Å, the NTD reaches only 10 Å. (**D**) Angular distribution of particles for final reconstruction. Each cylinder is proportional to the number of particles for that view.

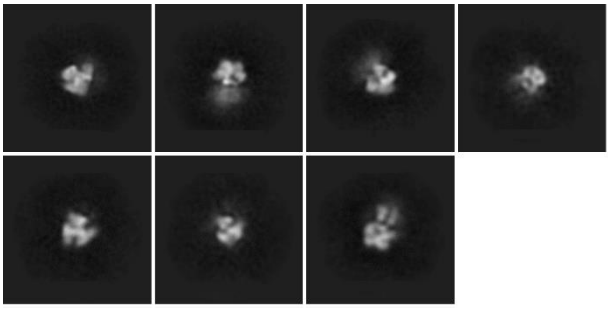

Figure S22. Some 2D Classifications of the *h*Lon_pCMF394 micrographs. As noted in the main text, the hexameric envelope of the ATPase-Protease "head" domain can be easily resolved, but is so flexible that alignment can be performed only using just the NTD alone. Thus the head appears unable to form a stable conformation.

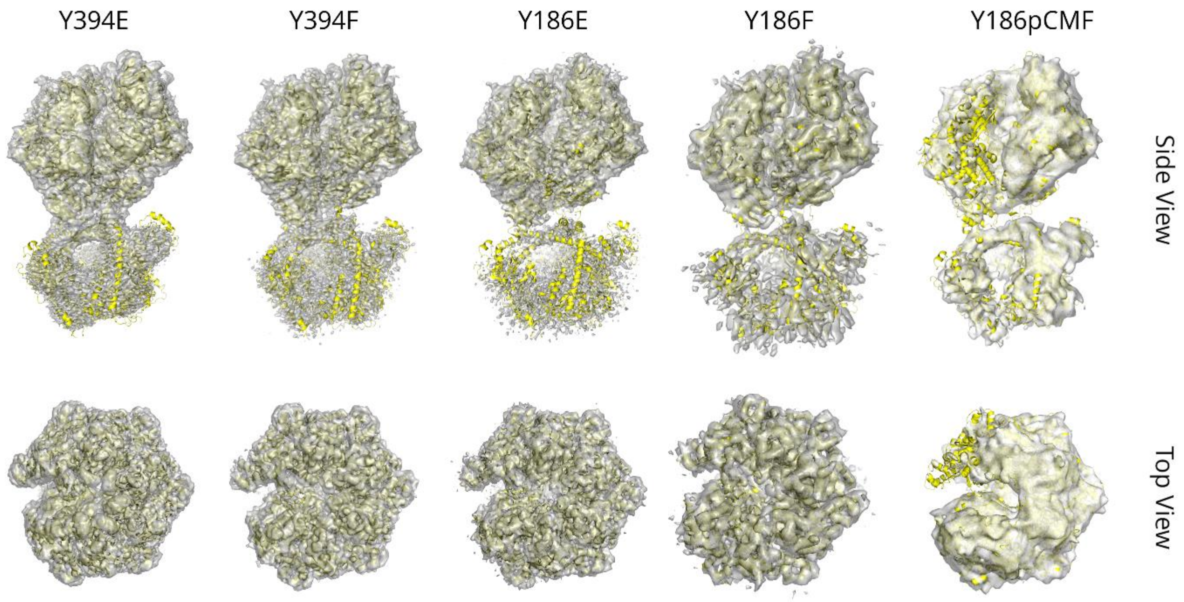

Figure S23. Cryo-EM potential maps of the Y394E, Y394F, Y186E, Y186F, and pCMF186 variants. Side and top views are shown. The maps are contoured at the following RMSD levels: Y394E, 4.0; Y394F, 4.0; Y186E, 5.5; Y186F, 6.0; pCMF186, 7.0.

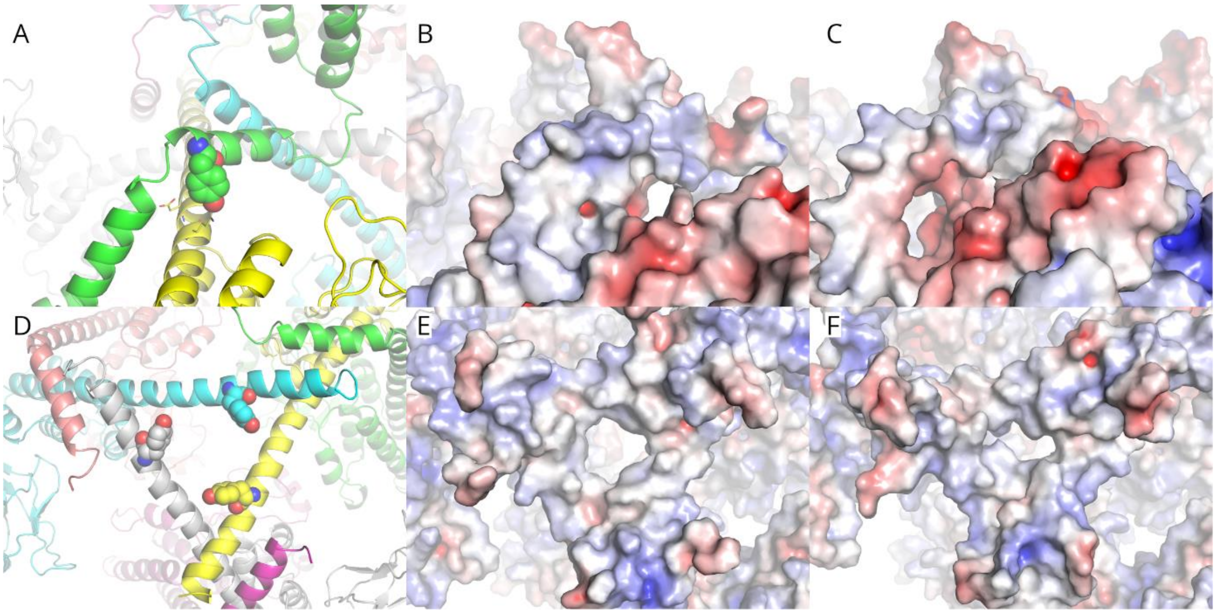

Figure S24. Additional electrostatic surfaces for Y394E and Y394F. (**A**) The position of Tyr394 in the three chains that place it outside of the triangular gate. It lies near the site where the long α-helix at the C-terminal end of the NTD bends and may make contacts between Lys368 and Glu371 from one of the subunits that comprise the triangular gate. (**B**) The electrostatic surface of the wild-type protein is slightly positively charged in this area, while the adjacent subunit it contacts is negatively charged. (**C**) The Y394E substitution makes this area slightly negatively charged, which may affect this contact. (**D**, **E**, **F**) The effect of the Y394E substitution on the electrostatic surface of the triangular gate separating the NTD and the ATPase-Protease domain. As in Figure 8, these surfaces were calculated over the NTD only. (**D**) The NTD gate from a wild-type *h*Lon structure (PDB ID 7OXO[3]); Tyr394 is shown as spheres. (**E**) The electrostatic surface of the wild-type and (**F**) the Y394F mutant. It can be seen that in both the wild-type protein and the Y394F mutant, the gate is neutral to positively charged. All electrostatic surfaces are shown at ±10 kT/e.

Table S9: Cryo-EM data collection and analysis.

	Y394E	Y394F	Y186E	Y186F	pCMF186
**Microscope**	FEI TiTan KRIOS	FEI Artica	FEI Artica	FEI Artica	FEI Artica
**Voltage (kV)**	300	200	200	200	200
**Camera**	Gatan K3	Gatan K2	Gatan K2	Gatan K2	Gatan K2
**Pixel size (Å)**	0.834	0.783	0.783	0.783	0.783
**Total dose (e^{-}/Å^2)**	40.0	40.0	40.0	40.0	40.0
**Exposure time (s)**	2.0	5.0	5.0	5.0	5.0
**Frames/exposure**	40	40	40	40	40
**Defocus range (µm)**	1–2	2–3	0.5–3	0.5–3	0.5–3
**No. micrographs**	12,393	1,376	1863	1154	1476
**No. particles**					
**initial**	591,879	102,680	150,666	274,052	460,619
**for 3D analysis**	367,678	34,462	58,404	50,342	23,915
**final**	325,507	33,120	39,948	37,657	23,915
**Resolution (Å)**					
**0.5 FSC**	3.23	4.54	4.33	9.18	16.7
**0.143 FSC**	2.88	3.94	3.76	7.23	8.50
**Refinement software**	PHENIX	PHENIX	PHENIX	PHENIX	PHENIX
**CC (mask)**	0.82	0.78	0.73	0.72	0.62
**Clash score**	5.61	10.75	10.49	8.43	8.26
**MolProbity score**	1.50	1.82	1.79	1.72	1.69
**Rotamers (%)**					
**poor**	0.07	0.05	0.02	0.0	0.0
**favored**	89.32	90.95	84.52	99.95	99.93
**Ramachandran plot (%)**					
**outliers**	0.00	0.00	0.00	0.00	0.00
**favored**	96.83	96.01	96.27	96.16	96.36
**Z-score**	0.36	0.26	0.01	0.16	0.26
**Bad bonds**	0	0	0	0	0
**Bad angles**	0	0	0	0	0
**PDB ID; EMDB ID**	8OJL; EMD-16915	8OKA; EMD-16923	8OM7; EMD-16970	8OVF; EMD-17213	8OVG; EMD-17214

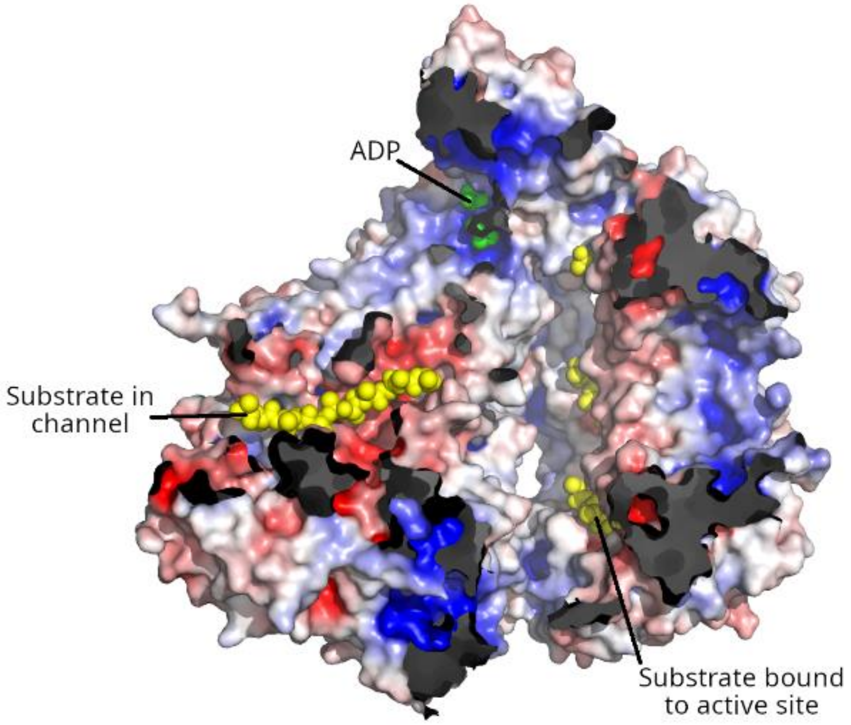

Figure S25. Electrostatic surface of the *h*Lon_K898A mutant bound to a substrate peptide. The model shown is 7P0M reported by Gesé *et al.*[4]. It shows the ATPase-Protease domain of *h*Lon bound to a substrate peptide in both the channel region and the active site of the protease active site. This view more clearly emphasizes the location of the substrate channel and clearly shows the negatively charged character of the entry tunnel, the neutral central cavity, and the positively charged exit pore. The active site region itself has a slight negative charged and the ATP binding site is strongly positively charged. The electrostatic surface is ramped at ±10 kT/e.

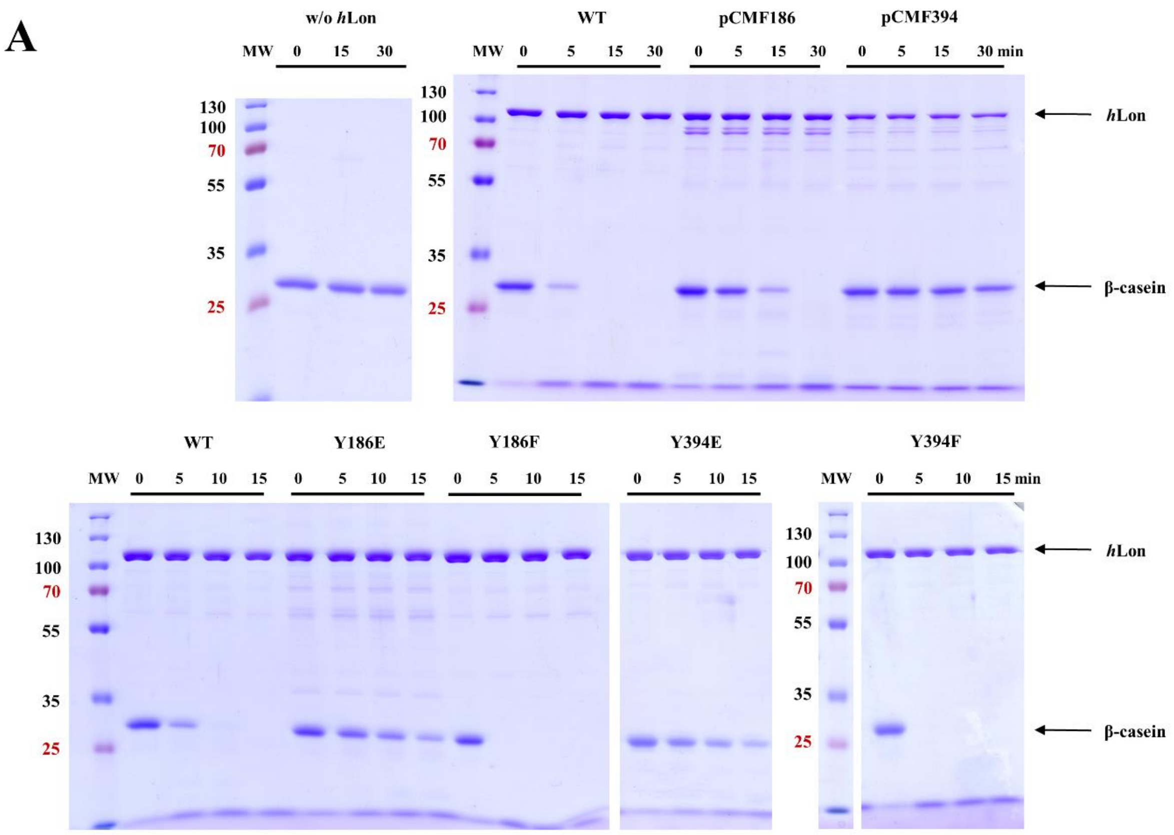

Figure S26. Protein substrates digested by the phosphorylated and phosphorylation-mimicking *h*Lon variants. The full-length gels shown in Figure 2A.

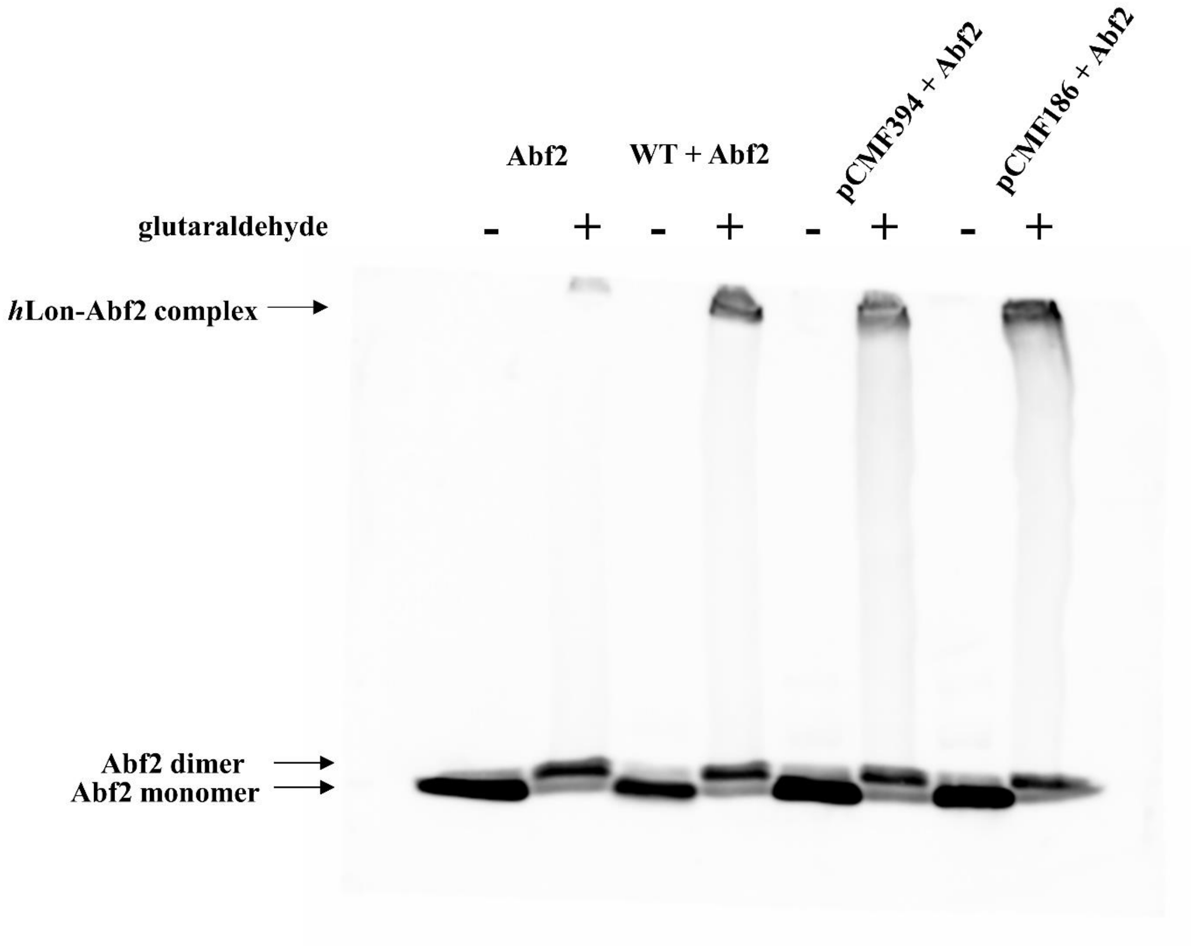

Figure S27. *h*Lon substrate-binding assay. The full-length unprocessed membrane from the western blot shown in Figure 4.

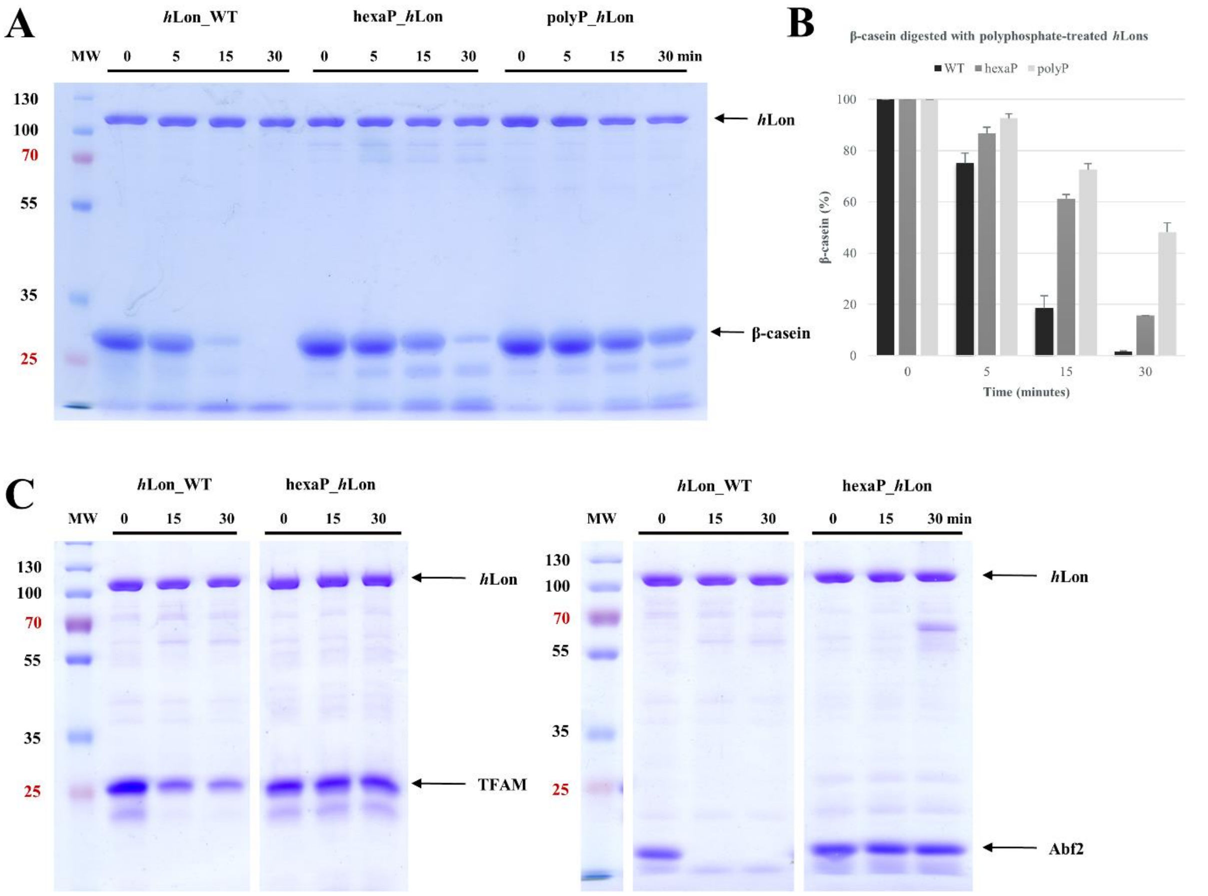

Figure S28. Protein substrates digested by hexa- and polyphosphate-treated *h*Lon. The full-length gels shown in Figure 5.

REFERENCES

1 Ambro, L. *et al.* Mutations to a glycine loop in the catalytic site of human Lon changes its protease, peptidase and ATPase activities. *FEBS J* **281**, 1784-1797, doi:10.1111/febs.12740 (2014).

2 Punjani, A., Rubinstein, J. L., Fleet, D. J. & Brubaker, M. A. cryoSPARC: algorithms for rapid unsupervised cryo-EM structure determination. *Nat Methods* **14**, 290-296, doi:10.1038/nmeth.4169 (2017).

3 Mohammed, I. *et al.* Catalytic cycling of human mitochondrial Lon protease. *Structure* **30**, 1254-1268 e1257, doi:10.1016/j.str.2022.06.006 (2022).

4 Gesé, G. V. *et al.* A dual allosteric pathway drives human mitochondrial Lon. *bioRxiv*, doi:10.1101/2021.06.09.447696 (2021).
